# Inhibition of Collagenase Q1 of *Bacillus cereus* as a Novel Antivirulence Strategy for the Treatment of Skin-Wound Infections

**DOI:** 10.1002/adtp.202100222

**Published:** 2022-01-15

**Authors:** Alaa Alhayek, Essak S. Khan, Esther Schönauer, Tobias Däinghaus, Roya Shafiei, Katrin Voos, Mitchell K.L. Han, Christian Ducho, Gernot Posselt, Silja Wessler, Hans Brandstetter, Jörg Haupenthal, Aránzazu del Campo, Anna K.H. Hirsch

**Affiliations:** Helmholtz Institute for Pharmaceutical Research Saarland (HIPS) Helmholtz Centre for Infection Research (HZI) 38124 Saarbrücken, Germany; Department of Pharmacy Saarland University, Saarbrücken Campus Campus E8.1, 66123 Saarbrücken, Germany; Leibniz Institute for New Materials (INM) Saarland University Campus D2 2, 66123 Saarbrücken, Germany; Department of Biosciences and Medical Biology Hellbrunner Str. 34 University of Salzburg Salzburg 5020, Austria; Leibniz Institute for New Materials (INM) Saarland University Campus D2 2, 66123 Saarbrücken, Germany; Helmholtz Institute for Pharmaceutical Research Saarland (HIPS) Helmholtz Centre for Infection Research (HZI) 38124 Saarbrücken, Germany; Department of Pharmacy Pharmaceutical and Medicinal Chemistry Saarland University Campus C2 3, 66123 Saarbrücken, Germany; Leibniz Institute for New Materials (INM) Saarl and University Campus D2 2, 66123 Saarbrücken, Germany; Department of Pharmacy Pharmaceutical and Medicinal Chemistry Saarland University Campus C2 3, 66123 Saarbrücken, Germany; Department of Biosciences and Medical Biology Hellbrunner Str. 34 University of Salzburg Salzburg 5020, Austria; Department of Biosciences and Medical Biology Hellbrunner Str. 34 University of Salzburg Salzburg 5020, Austria; Department of Biosciences and Medical Biology Hellbrunner Str. 34 University of Salzburg Salzburg 5020, Austria; Helmholtz Institute for Pharmaceutical Research Saarland (HIPS) Helmholtz Centre for Infection Research (HZI) 38124 Saarbrücken, Germany; Leibniz Institute for New Materials (INM) Saarland University Campus D2 2, 66123 Saarbrücken, Germany; Chemistry Department Saarland University 66123 Saarbrücken, Germany; Helmholtz Institute for Pharmaceutical Research Saarland (HIPS) Helmholtz Centre for Infection Research (HZI) 38124 Saarbrücken, Germany; Department of Pharmacy Saarland University, Saarbrücken Campus Campus E8.1, 66123 Saarbrücken, Germany

**Keywords:** antibiotic resistance, *Bacillus cereus*, collagenase, pathoblocker, virulence factors

## Abstract

Despite the progress in surgical techniques and antibiotic prophylaxis, opportunistic wound infections with *Bacillus cereus* remain a public health problem. Secreted toxins are one of the main factors contributing to *B*. *cereus* pathogenicity. A promising strategy to treat such infections is to target these toxins and not the bacteria. Although the exoenzymes produced by *B*. *cereus* are thoroughly investigated, little is known about the role of *B*. *cereus* collagenases in wound infections.

In this report, the collagenolytic activity of secreted collagenases (Col) is characterized in the *B*. *cereus* culture supernatant (csn) and its isolated recombinantly produced ColQ1 is characterized. The data reveals that ColQ1 causes damage on dermal collagen (COL). This results in gaps in the tissue, which might facilitate the spread of bacteria. The importance of *B*. *cereus* collagenases is also demonstrated in disease promotion using two inhibitors. Compound 2 shows high efficacy in peptidolytic, gelatinolytic, and COL degradation assays. It also preserves the fibrillar COLs in skin tissue challenged with ColQ1, as well as the viability of skin cells treated with *B*. *cereus* csn. A *Galleria mellonella* model highlights the significance of collagenase inhibition in vivo.

## 1 Introduction

*Bacillus cereus* (*B*. *cereus*) is a widely distributed Gram-positive bacterium. This bacterium is the major cause of emetic and diarrheal food poisoning worldwide, but also associated with serious opportunistic non-gastrointestinal-tract infections.^[1,2]^ Moreover, it is able to cause wound infections.^[1,3,4]^ Like many pathogenic bacteria, *B*. *cereus* is currently evolving multi-drug resistance,^[5–7]^ which narrows the choice of possible treatments and consequently increases economic costs, morbidity, and mortality rates.^[8–10]^ To overcome this therapeutic crisis, the development of new antibiotics will not produce lasting success, but alternative strategies need to be employed to cope with resistance development.^[11]^ To combat the emergence of resistance, the development of antivirulence agents targeting the pathogenicity of bacteria rather than their viability, has gained major interest.^[11–13]^ These agents specifically block the virulence factors involved in bacterial invasion and colonization of the host.^[14]^ This reduces the selection pressure for drug-resistant mutants and provides a window of opportunity for the host immune system to eliminate the bacteria.^[7,11,13]^ The pathogenicity of *B*. *cereus* arises from the production and dissemination of tissuedestructive exoenzymes such as hemolysins, phospholipases, and proteases.^[1,15,16]^ It is believed that these exoenzymes assist in maintaining the infection, allowing the bacteria to reach multiple sites in the body and to evade the immune system. There have been only few studies to support the idea of *Bacillus* exoenzymes contributing to the pathology of wound infections and little evidence to elucidate the direct role of specific toxins during the infection.^[17,18]^

The skin is the largest and most exposed of all human organs and, therefore, most prone to injury.^[19]^ The dermal layer makes up 90% of the skin structure.^[20]^ The architecture and integrity of the dermis are maintained by COL. COL I, II, and III are predominant in the extracellularmatrix (ECM) of the skin.^[21,22]^ COL fibers are supramolecular structures, COL molecule is made up by regular packing of three supertwisted alpha helices.^[21,22]^ The individual alpha chains consist of a repeated three amino acid motif (Glycine-X-Y), with X-Y often being proline (28%) and hydroxyproline (Hyp) (38%).^[21,22]^ Because of its highly intertwined structure and high content of specific amino acids (i.e., Glycine-X-Y),^[23]^ fibrillar COLs resistmost proteases and can be degraded only by certain types of mammalian or bacterial collagenaseswith unique specificities to degrade COL.^[21,24,25]^

Bacterial wound infection is a public health problem occurring when bacteria adhere to an impaired skin.^[26,27]^ After the initial local colonization, bacteria can potentially invade into deeper tissues with the help of necrotic virulence factors such as collagenases.^[26,28]^ By degrading the structural COL scaffold of the ECM at multiple sites, bacterial collagenases assist the bacteria in invading the tissue.^[29,30]^ Bacterial collagenases belong to the zinc metalloprotease family M9.^[29]^ They harbor a collagenase unit, which is accompanied by accessory domains involved in substrate recognition and COL swelling.^[29]^ To date, only a few collagenase-secreting bacterial genera (e.g., *Bacillus*, *Clostridium*, and *Vibrio*) have been identified. *Clostridium* collagenases such as ColH and ColG are the best characterized ones.^[29]^ *Bacillus* collagenases have received less attention. Their contribution to wound infections however is assumed to be a main factor in the woundinvasion stage.

Here, we report on the establishment of a simple pre-clinical ex vivo pig-skin model to evaluate the effect of COL degradation by *B*. *cereus* in the skin. Our results showed that the model *B*. *cereus* collagenase ColQ1 degrades the dermal fibrillar COLs and confirmed it as a promising for drug target. Using two small molecules, which we had recently described as inhibitors of the collagenase ColH (produced by *Clostridium histolyticum*)^[31]^ and the elastase LasB (produced by *Pseudomonas aeruginosa*),^[32]^ we could substantiate that these inhibitors also inhibit *B*. *cereus* collagenase activity. Indeed, we found that these compounds were able to protect the integrity of the dermal COL in an ex vivo pigskin model treated with recombinant ColQ1, confirming their potency as broad-spectrum inhibitors of bacterial collagenases, as suggested earlier by Schönauer et al.^[33]^ Moreover, these compounds reduced in vitro cytotoxic effects of the *B*. *cereus* csn, containing various collagenases, toward fibroblast and keratinocyte cell lines, restored their morphology, and improved their adhesion. The toxicity of *B*. *cereus* csn and ColQ1 was verified in vivo in *Galleria mellonella* larvae. Furthermore, we showed that treatment with collagenase inhibitors significantly improved their survival rate.

## 2 Results and Discussion

### 2.1 *B*. *cereus* csn and Recombinant *B*. *cereus* ColQ1 Act as Collagenolytic Agents

To study the effect of bacteria-derived collagenase on COL degradation in skin wounds, we used the recombinant collagenase unit of ColQ1 (Uniprot: B9J3S4)^[34]^ and the csn of *B*. *cereus* ATCC 14 579^[35]^ to challenge our skin model. ColQ1 was selected as a model *Bacillus* collagenase to study the isolated effect of this virulence factor in a skin wound setting. ColQ1 is a close homologue of ColA of *B*. *cereus* ATCC 14 579 (Uniprot: Q81BJ6) and similarly to ColA, it displays a remarkably high peptido- and collagenolytic activity compared to clostridial collagenases.^[34]^ Both enzymes share an overall sequence identity of 72% and a similarity of 84%. Sequence conservation is higher within the collagenase unit, i.e., the catalytic core of the enzyme, increasing to 79% and 89%, respectively.^[34,36]^ Proteolytic activity of ColQ1 and of *B*. *cereus* csn (which represents amore complex source of COL-degrading factors)^[34,36]^ were validated in an in vitro peptidolytic assay using a custom-made collagenase-specific quenched fluorescence substrate.^[33]^ The csn of *B*. *cereus* showed peptidolytic activity that could be completely abrogated by the addition of 20mM EDTA and was only marginally affected by serine and cysteine protease inhibitors, consistent with its metalloprotease mechanism (Figure S1, Supporting Information). The peptidolytic activity of the csn determined in the presence of serine and cysteine protease inhibitors was comparable to the activity of 0.9 ± 0.1 × 10^−9^ M of recombinant collagenase unit of ColQ1. These results were determined based on a standard curve that was generated using recombinant ColQ1 (Figure S2, Supporting Information).

### 2.2 *B*. *Cereus*-Induced COL Degradation Quantified in an Ex vivo Pig-Skin Model

**Figure 1:**
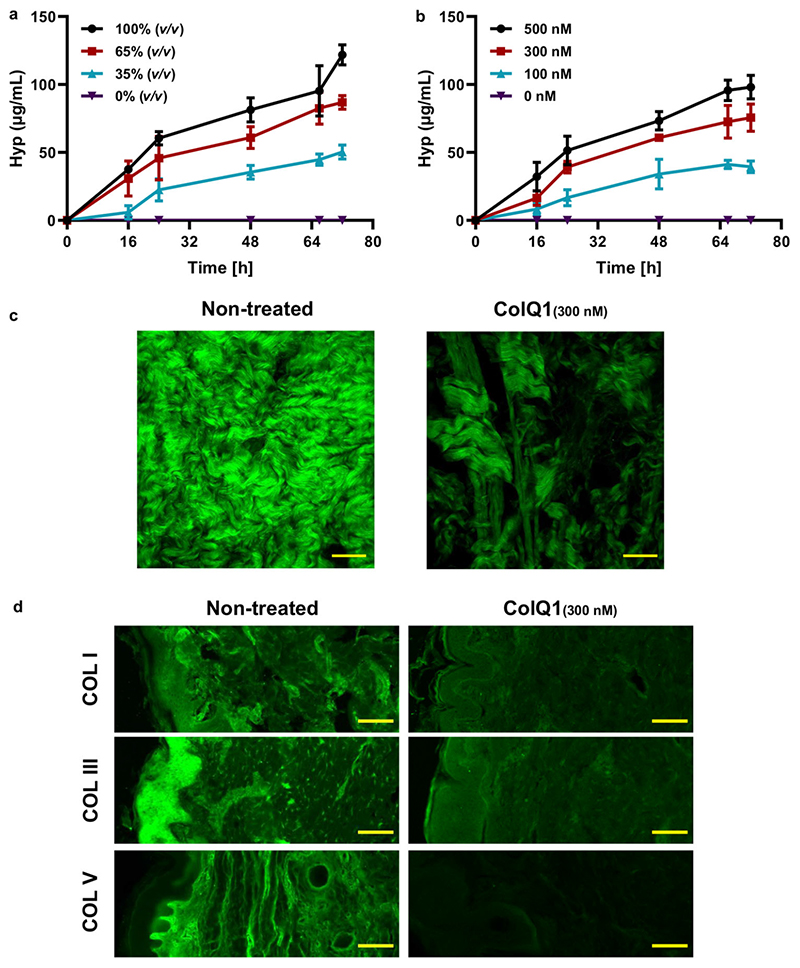
The effect of ColQ1 on dermal COL of pig-skin. **a,b)** Quantification of Hyp release over time after treatment with different concentrations of a) *B*. *cereus* csn (0−100% v/v) and b) ColQ1 (0−500 × 10^−9^ M). This graph contains data adapted from our previous publication.^[38]^ **c)** Confocal SHG Z-stack images of the COL structure in skin dermal region that was non-treated or treated with 300 × 10^−9^ M ColQ1. **d)** *B*. *cereus* ColQ1 (300 × 10^−9^ M) degraded the fibrillar COLs, immunostaining of non-treated and ColQ1 treated skin with COL antibodies (COL I, III, and V). COL: collagen, Hyp: hydroxyproline, *B*. *cereus*: *Bacillus cereus*, csn: culture supernatant, SHG: second harmonic generation. Data point represents mean value ± standard deviation (*n* = 3). Scale bar: 100 μm for SHG images and immunostained images. Bright-field and DAPI images of the immunostained non-treated and ColQ1-treated tissue are shown in Figure S4, Supporting Information.

To analyze the collagenolytic activity of *B*. *cereus* csn and ColQ1 during wound infection, an ex vivo pig-skin model of *B*. *cereus* infection was established.^[38]^ For this purpose, porcine ear skin biopsy punches were treated with different concentrations of *B*. *cereus* csn (35%, 65%, and 100% v/v) or ColQ1 (100 × 10^−9^, 300 × 10^−9^, and 500 × 10^−9^ M) to simulate COL matrix degradation after infection with *B*. *cereus*. The release of hydroxyproline (Hyp) was used as a biomarker for COL breakdown.^[39,40]^ While we did not observe Hyp release in non-treated skin preparations, a significant release was detected in skin treated with various concentrations of *Bacillus* csn and ColQ1 (Figure 1a,b). In detail, incubation with 35% (v/v) of the csn led to an increase ofHyp levels to 22 ± 6 μgmL^−1^ release of Hyp after 24 h, and 100% (v/v) csn, Hyp levels rendered 60 ± 4 μgmL^−1^ (Figure 1a) Hyp in the supernatant. As we showed before,^[38]^ treatment with 100 × 10^−9^ or 500 × 10^−9^ M of the enzyme led to 16 ± 6 μgmL^−1^ and 51 ± 10 μgmL^−1^ Hyp release after 24 h incubation, respectively (Figure 1b). Longer incubation times led to largerHyp concentrations until a plateau value was reached. These data confirm that ColQ1 exhibited an effect on collagen degradation that was comparable to the csn. To analyze the collagenase-specific effects, we focused on ColQ1 in the following ex vivo studies.

We visualized the loss of matrix COL of the skin tissue challenged with ColQ1 using SHG imaging. This method allows a label-free imaging of the COL fibers.^41^ The confocal SHG Z-stack images of non-treated skin showed COL structures with the characteristic wave-like morphology of dermal COL^[42]^ (Figure 1c). This morphology is essential for elastic integrity and it provides the biomechanical prerequisites necessary to sustain the shape and strength of the skin tissue.^[20,43,44]^ In contrast, skin treated with 300 × 10^−9^ M ColQ1 showed a lower SHG signal and large gaps between the COL structures (Figure 1c). Higher ColQ1 concentrations resulted in fragile tissue samples and lower ColQ1 concentrations did not show a significant collagenolytic effect (data not shown). Therefore, a concentration of 300 × 10^−9^ M ColQ1 was chosen for further experiments.

Our evaluation of both COL structure and the released Hyp showed that *B*. *cereus* collagenases have a highly destructive effect on native COL in skin. Based on the disruptive effect of *B*. *cereus* collagenase on the collagenmatrix, we hypothesize, that collagenolytic activities diminish skin tissue integrity and thus aid passage of the bacteria to deeper dermal layers in settings of wound infection.

### 2.3 ColQ1 Targets Fibrillar COLs in the Dermis

The skin dermis and hypodermis are rich in COL I, which forms heterotypic structures with other COLs such as III and/or V.^[45]^ To test the ability of ColQ1 to target these fibrillar COLs in skin, immunostaining of skin samples after the treatment was performed with antibodies against COL I, III, and V followed by epifluorescence imaging of the stained tissue. Non-treated samples showed strong signals for COL I in the dermis, COL III in the epidermis, and around cellular components of the dermis, and COL V in the basal and dermal layers (Figure 1d). Upon treatment with ColQ1 a moderate reduction in the signal of all three fibrillar COLs (i.e., Col I, III, and V) was observed (Figure 1d). These data indicate that ColQ1 is targeting fibrillar COL subtypes enriched in the dermal region. The effect of ColQ1 on fibrillar COLs can be explained by the tertiary and primary structure of the substrate. Fibrillar COLs are mainly composed of one large triple-helical domain (e.g., COL I: 96%) with (Gly-X-Y) tripeptide repeats.^[45]^ The active site sequence specificity of bacterial collagenases is perfectly adapted to this tripeptide motif, as it has been shown for clostridial collagenases.^[46]^

### 2.4 Collagenase Inhibitors Neutralize the Collagen Degradation Effect of *B*. *cereus* Collagenases In vitro

To study whether we could inhibit ColQ1 with small molecules, we investigated two previously described inhibitors of bacterial metalloproteases. Compound **1** is one of the first reported ColH inhibitors (IC_50_ = 7 × 10^−6^ m) being stable and selective over several human metalloproteases.^[31]^ Compound **2** is amoderately active LasB inhibitor (IC_50_ = 17.3 × 10^−6^ m)^[32]^ and was a hit in a virtual screening study performed on the active site of ColH.

**Figure 2:**
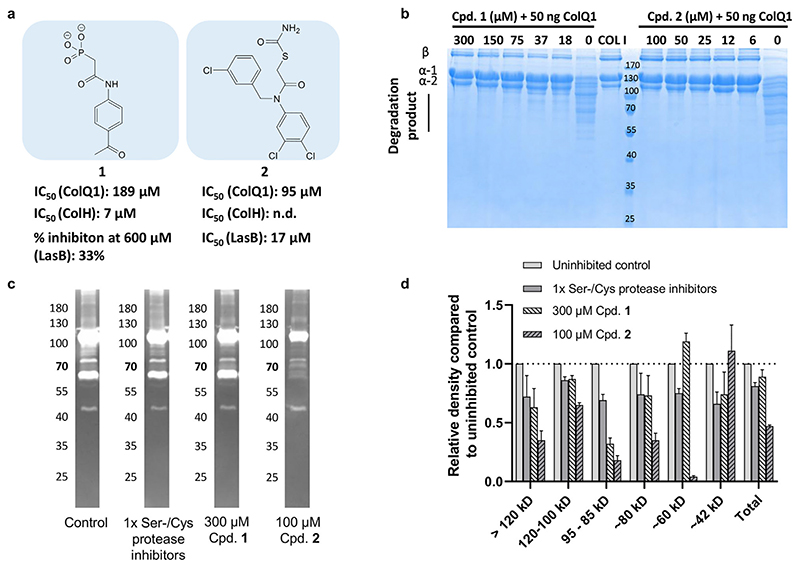
Inhibition of ColQ1 and the collagenase of *B*. *cereus* csn by compounds **1** and **2** in a collagenase-specific peptidic and a gelatinolytic assay. **a)** Chemical structures of compounds **1** and **2** and the calculated IC_50_ value in the FRET-based ColQ1, ColH,^[31]^ and LasB^[32]^ inhibition assay. **b)** Effect of ColQ1 inhibitors on the cleavage of COL I after challenge with 50 ng of ColQ1. **c,d)** Effect of compounds **1** and **2** on *B*. *cereus* csn monitored by c) gelatin zymography. The gelatin-degradation assay was performed in the presence of inhibitors or the buffer control. Due to limited solubility in the reaction buffer, compound **2** could only be tested at 100 × 10^−6^ m compared to 300 × 10^−6^ m of compound **1**. **d)** Densitometric analysis of gelatin zymography shown in (c). Image analysis was performed with Image Studio Lite v5.2 software (Li-Cor Biosciences, USA). *B*. *cereus*: *Bacillus cereus*, csn: culture supernatant, COL: collagen, n.d.: not determined.

Using a FRET-based peptidolytic assay with a collagenasespecific substrate as well as a COL cleavage assay with the natural triple-helical substrate of collagenases (i.e., COL I), the impact of these two inhibitors on ColQ1 activity was measured in vitro. The FRET-based assay confirmed that compounds **1** and **2** inhibit ColQ1 with IC_50_ values of 183 ± 7 × 10^−6^ m^[31]^ and 95 ± 4 × 10^−6^ m, respectively (Figure S3, Supporting Information; Figure 2a). In addition, the COL cleavage assay demonstrated a full collagenase inhibition with protection of the structural integrity of COL I at 75 × 10^−6^ and 6 × 10^−6^ m with compounds **1** and **2**, respectively (Figure 2b).

To further investigate the activity of compounds **1** and **2**, we tested them on the *B*. *cereus* csn, which contains a heterogeneous mixture of ColA isoforms and other collagenase homologs. The *B*. *cereus* csn was treated with 1.83 mM (10 × IC_50_) of compound **1**. The FRET-based assay revealed that the proteolytic activity furnished by the csn could be reduced by 84 ± 2% compared to the uninhibited control (Figure S1, Supporting Information). Due to the low solubility of compound **2** under assay conditions, compound **2** could only be tested at a concentration of 95 × 10^−6^ m (1 × IC_50_). Remarkably, this concentration led to a decrease in the proteolytic activity of 57 ± 7% (Figure S1, Supporting Information). The positive control (20 mM EDTA) completely inhibited substrate turnover, while an inhibitor cocktail specific for serine and cysteine proteases reduced the total activity by only 14 ± 7% (Figure S1, Supporting Information).

We could qualitatively confirm the inhibitory effect of compounds **1** and **2** on the *B*. *cereus* csn using gelatin zymography (Figure 2c,d). For this, the csn was separated by electrophoresis and then subjected to an in-gel activity assay. Gelatinolytically active species were detected by the degradation of denatured COL I that had been co-polymerized with the polyacrylamide matrix of the SDS-PAGE gel, visible as white bands in the zymogram. Similar to previous reports,^[35,48]^ it revealed the presence of various gelatinolytically active species in the csn of *B*. *cereus* most prominently at amolecular weight of approx. 115 kDa and smaller. The zymogram performed in presence of i) serine and cysteine protease inhibitors, ii) compound **1**, and iii) compound **2** showed a selective reduction of the gelatinolytic activities in all cases (Figure 2c). In particular, the high molecular weight species corresponding to full-length ColA and C-terminally truncated ColA species in the range of 120−80 kDa, as identified before by Abfalter et al.,^[35]^ were inhibited by compounds **1** and **2** (Figure 2c).

In all in vitro assays, compound **2** was more active than compound **1**. Both compounds not only inhibit ColH, LasB, and ColQ1, as reported previously,^[31,32]^ but also demonstrated an inhibitory effect on gelatinases of *B*. *cereus* csn. We have previously reported a similar broad-spectrum inhibition of *Bacillus* and *Clostridium* collagenases in in vitro assays for closely related compounds,^[33,38]^ which might be beneficial in wound infections colonized by multiple bacterial genera.^[28]^

### 2.5 Docking Studies with Bacterial Collagenases Rationalize Differences in Inhibitory Potency

The observed difference in efficacy between compounds **1** and **2** can be rationalized based on the binding mode of both compounds to bacterial collagenases. For this purpose, molecular docking was performed using the crystal structure of the peptidase domain of ColH as target that had been determined at a resolution of 1.87 Å. The crystal structure of the homologue ColH was chosen, as there are to date no high-resolution crystal structures from a *B*. *cereus* collagenase available to ensure reliable docking results. The peptidase domain of ColH shares 74% and 73% sequence similarity with the peptidase domains of ColA and ColQ1 from *B*. *cereus*, respectively, and the sequence and topology of the active sites are highly conserved.^[34,35]^ Since docking to metallo-proteins is non-trivial in drug design, AutoDock Vina v1.2.2^[48]^ and the Molecular Forecaster suite^[49]^ were both evaluated for this end and their performance judged by their ability to generate poses that comply with standard atom-to-zinc distances and zinc-binding geometries.^[50]^ Following this criterion, theMolecular Forecaster suite was used for the final docking of compounds **1** and **2** to ColH.

**Figure 3:**
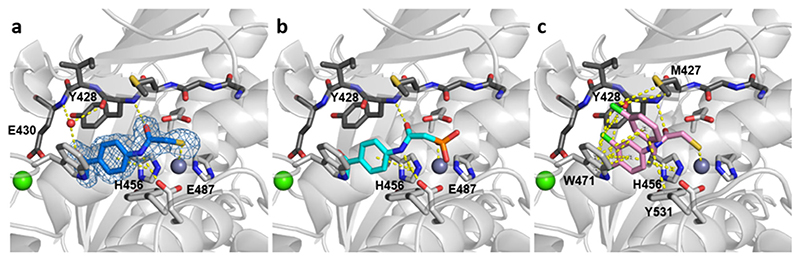
Comparison of the crystallized complex of the N-aryl mercaptoacetamide compound with the docking poses of compounds **1** and **2** in the active site of ColH. Comparison of the crystallized complex of the *N*-aryl mercaptoacetamide compound with the docking poses of compounds **1** and **2** in the active site of ColH. **a)** Close-up view of the active site in ball-and-stick representation. The co-crystallized inhibitor (blue) is shown in sticks with the maximum likelihood weighted 2Fo − Fc electron density map contoured at 1*σ*. Top docking poses of compounds **1 b)** and of **2 c)** in the active site of ColH. The catalytic zinc ion (dark gray), calcium ion (green), and water molecule (red) are shown as spheres. The edge strand formed by Gly425 to E430 is shown in dark gray sticks. The figures were prepared using the PyMOL Molecular Graphics System.

As expected, we found that the best docking pose for compound **1** showed a similar binding mode as was determined for the*N*-arylmercaptoacetamide ligand in the complex crystal structure with ColH^[33]^ (Figure 3a,b). Both compounds share the same *N*-aryl backbone, but differ in their zinc-binding group. Instead of the thiolate sulfur atom of the mercaptoacetamide compound, the phosphonate oxygen atom of compound **1** is predicted to coordinate the catalytic zinc ion (2.1 Å). The amide oxygen and nitrogen atoms form a hydrogen bond with the main-chain amide nitrogen atom of Tyr428 and the carbonyl oxygen of Glu487, respectively, while the aryl ring of compound **1** is involved in a *π-π*-stacking interaction with the imidazole ring of His459 (3.9 Å). In contrast to compound **1**, compound **2** has a different, much larger molecular backbone, but shares the same thiol prodrug moiety with the co-crystallized *N*-aryl mercaptoacetamide,^[33]^ i.e., a thiocarbamate group. Similarly to the *N*-aryl mercaptoacetamide, we found that the deprotonated sulfur atom of compound **2** can coordinate the active-site zinc cation (2.3 Å), while the amide oxygen forms a hydrogen bond with the main-chain nitrogen atom of Tyr428 (Figure 3c). The active site of ColH can accommodate the two aromatic moieties of compound **2** in the non-primed side via a network of *π-π*-stacking interactions involving His456, Trp471, and Tyr531, which is supported by a parallel network of *π*-alkyl and *π*-sigma interactions via the chlorine substituents with Tyr428, Trp471, and Met427. This extensive system of *π*-interactions found by the docking experiment anchors compound **2** firmly into the active site in-between the upper and lower subdomains of the peptidase domain and itmight explain the observed higher efficacy of compound **2** compared to compound **1** that lacks this dense interaction network.

### 2.6 Compounds 1 and 2 Inhibit the Collagenolytic Activity of ColQ1 in an Ex vivo Pig-Skin Model

**Figure 4:**
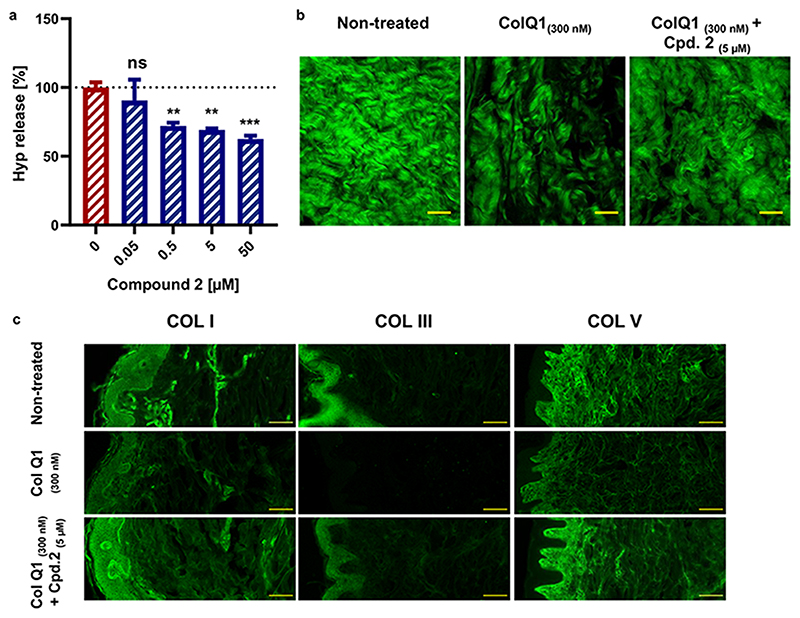
Compound **2** suppressed the collagenolytic effect of ColQ1 ex vivo in skin tissue. **a)** Dose-dependent effect of compound **2** quantified by Hyp release assay. **b)** Confocal SHG images showed an improved COL signal with 5 × 10^−6^ m of compound **2** (tissue challenged with 300 × 10^−9^ M ColQ1) compared with 300 × 10^−9^ M ColQ1 without inhibitor. **c)** Immunostaining of fibrillar COLs of the non-treated skin and treated with ColQ1 with or without compound **2**. Statistical analysis was performed with one-way ANOVA and statistical significance was analyzed by Tukey test. Significance was calculated by comparing non-treated versus treated tissue with compound **2** (mean ± SD, *** *p* ≤ 0.001, ** *p* ≤ 0.01). Hyp: hydroxyproline, COL: collagen, SHG: second harmonic generation. Scale bar: 100 μm for SHG images and 100 μm for the immunostained images.

As compounds **1** and **2** suppressed ColQ1 activity in vitro, we furthermore tested their effects on collagenase activity in the skin model. Different concentrations of compounds **1** (50−400 × 10^−6^ m) and **2** (0.05-50 × 10^−6^ m) based on their activity in the different in vitro assays along with 300 × 10^−9^ M ColQ1 were used. Non-treated and ColQ1-treated samples were used as controls. After one day of incubation, we quantified the release of Hyp and visualized the dermal COL in the skin tissue using SHG and epifluorescencemicroscopic techniques. Overall, compound **1** resulted in a reduction in Hyp release in a concentrationdependent manner as we had shown previously (Figure S6, Supporting Information).^[31]^A concentration of 300 × 10^−6^ mof compound **1** was selected for further analysis. Addition of compound **2** at concentrations between 5 and 50 × 10^−6^ m caused a reduction in the release of Hyp by 35% and 48%, respectively (Figure 4a). Based on these data, a concentration of the inhibitorymolecule **2** of5 × 10^−6^ m was chosen for further analysis.

Next, we performed SHG imaging of samples treated with compounds **1** or **2**. The ability of these molecules to reduce the ColQ1-mediated degradation of matrix COL fibers was confirmed compared to the ColQ1-treated control. A higher density of collagen fibers was observed in the presence of both compounds, similar to the morphology of non-treated skin (Figure 4b; Figure S6, Supporting Information). Further experiments were carried out to investigate which COL types (I, III, and V) are protected in presence of compounds **1** and **2**. We performed epifluorescence imaging with the tissue treated with 300 × 10^−9^ M ColQ1 and 300 × 10^−6^ m compound **1** or 5 × 10^−6^ m compound **2**. Both compounds led to a higher-intensity signal for COL I, V, and III when compared to the signal of the ColQ1-treated skin control (Figure 4c; Figure S6, Supporting Information). Overall, the results from the ex vivo skin model support the previous results on Hyp release and confirm that ColQ1 inhibition prevents degradation of fibrillar COL. Moreover, the findings underline the higher efficacy of compound **2** compared to compound **1** that had initially been observed in our in vitro assays.

### 2.7 Collagenase Inhibitors Reduce the Cytotoxic Effect of *B*. *cereus* csn on Human Skin Cell Lines

We further investigated whether the *B*. *cereus* csn has a cytotoxic effect on skin cells and whether this effect could be inhibited with collagenase targeting pathoblockers (i.e., compounds **1** and **2**). For this purpose, normal human dermal fibroblasts (NHDF) and human epidermal keratinocytes (HaCaT). were chosen due to their ability to produce fibrillar COLs and their roles during wound healing.^[51]^

These cells were exposed to different concentrations of *B*. *cereus* csn (0−15% v/v). The cytotoxic effect of the csn was evaluated by assessing the viability using a colorimetricMTT assay^[52]^ and live/dead staining,^[53]^ followed by visualization with epifluorescence microscopy.

A reduction in the viability of the cells was observed depending on the concentration of the *B*. *cereus* csn (Figure S7, Supporting Information). This cytotoxic effect increased slightly with incubation time of 24 h to 48 h (Figure S7, Supporting Information). The csn appeared more toxic forHaCaT cells than forNHDFcells (Figure S7, Supporting Information). The difference between the toxicity against HaCaT and NHDF cells might be due to the protective effect of fibroblasts provided by its high collagen contents, which help them to maintain the structure of the dermal layer.^[54]^ Bright-field images showed a strong detachment of cells, rounding, and shrinkage in both cell lines (example for NHDF cells is shown in Figure S8, Supporting Information, indicating apoptosis).

**Figure 5:**
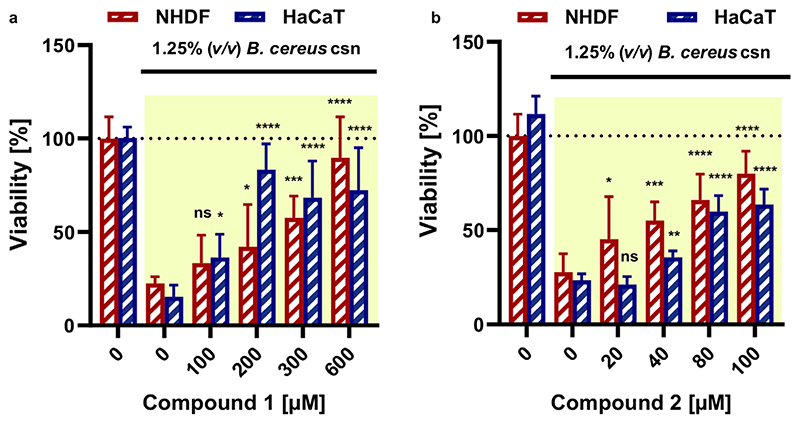
Compounds **1** and **2** maintained the viability of skin cells upon treatment with 1.25% (v/v) of *B*. *cereus* csn. Cell viability calculated after performance of an MTT assay for the cells challenged with *B*. *cereus* csn with **a)** compounds **1** and **b) 2**. The data in yellow background indicate cells treated with the csn.Statistical analysis was performed with one-way ANOVA and statistical significance was analysed by Tukey test. Significance was calculated by comparing non-treated versus treated cells with compound **1** and **2** (mean ± SD, **** *p* ≤ 0.0001, *** *p* ≤ 0.001, ** *p* ≤ 0.01, * *p* ≤ 0.05, and ns: non-significant). B. cereus: *Bacillus cereus*, csn: culture supernatant.

To demonstrate the inhibitory effect of compounds **1** and **2** in subsequent experiments, we used 1.25% (v/v) of the *B*. *cereus* csn, due to the prominent cytotoxic effects observed at this concentration in both NHDF and HaCaT cell lines. Cell viability was dose-dependent, but a significant rescue of viability (80 ± 20% and 70 ± 25%) was observed at 600 × 10^−6^ and 100 × 10^−6^ m of compounds **1** and **2,** respectively, in both NHDF and HaCaT cell lines (Figure 5). The live/dead staining results were consistent with the MTT data and showed an increase in the number of viable relative to dead cells (Figure S9, Supporting Information). Both compounds showed high viability at high concentration, which confirms their activity against the collagenase and its isoforms and maybe against other virulence factors. The property of these compounds to restore the viability of NHDF cells is important for a therapeutic context, since fibroblasts are active depositors of matric proteins in connective tissues in the processes of wound closure.^[51]^ Also, keratinocytes play an important role during wound healing, as they fill the gaps in the wound and produce proinflammatory mediators once pathogen invasion starts.^[55,56]^ The protection of both cell types by collagenase inhibitors is promising, as their cross-talk is fundamental to assure wound healing and hemostasis.^[57]^ Thus, collagenase inhibitorsmight serve as promising therapeutic agents in the future not only to stop bacterial dissemination but also to accelerate the immune response and subsequently accelerate the woundhealing process.^[58,59]^

### 2.8 Collagenase Inhibitors Diminish the Virulence Activity Induced by ColQ1 and *B*. *cereus* csn on *Galleria mellonella* Larvae

To examine the virulence of the *B*. *cereus* collagenase ColQ1 or csn and their inhibition in a simple in vivo model, *Galleria mellonella* larvae were used. This model is accepted as an alternative to murine models in microbial infection research due to its ease to obtain and use without elaborate equipment and ethical considerations.^[60]^ Moreover, the mechanisms of the innate immune system are closely related to those of the _mammals._^[61,62]^

**Figure 6:**
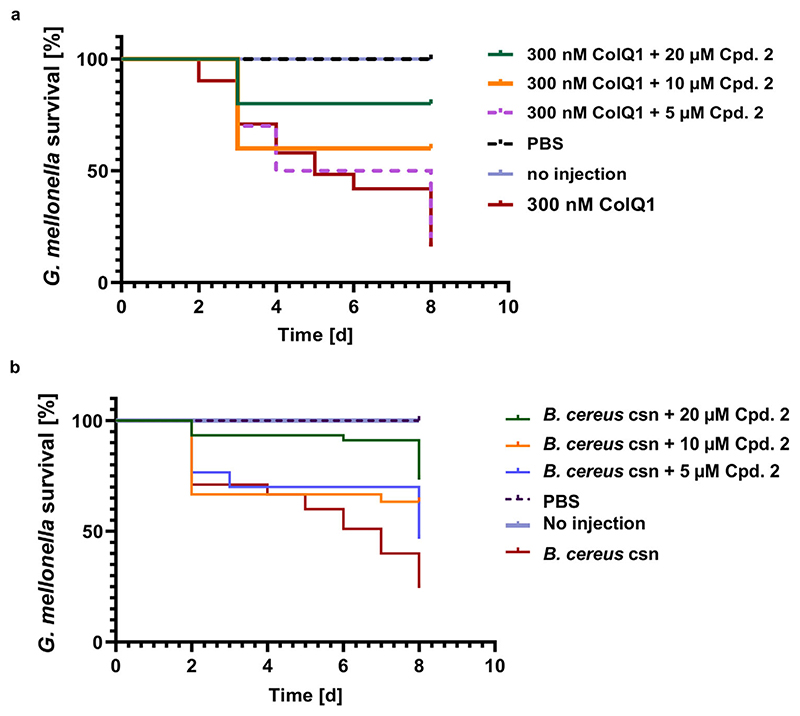
Kaplan−Meier survival analysis of larvae treated with *B*. *cereus* csn with and without compound **2**. **a)** Survival analysis of larvae treated with 300 × 10^−9^ M ColQ1 and with various concentrations (5 × 10^−6^−20 × 10^−6^ m) compound **2**. **b)** The improvement in the survival of larvae challenged with 100% (v/v) *B*. *cereus* csn and various concentrations of compound **2** (5 × 10^−6^−20 × 10^−6^ m). The statistical difference between groups treated with 20, 10, and 5 × 10^−6^ m of compound **2** and treated with only 300 × 10^−9^ M ColQ1 is *p* 0.0001, *p* = 0.0042, and *p* = 0.5800, sequentially (log-rank). The statistical difference between groups treated with 20, 10, and 5 × 10^−6^ m of compound **2** and treated with only 100% (v/v) *B*. *cereus* csn is *p* 0.0001, *p* = 0.0052, and *p* = 0.034, respectively. The survival rate for the larvae treated with compound **2** in PBS was 100%. *B*. *cereus*: *Bacillus cereus*, csn: culture supernatant.

To explore the effect of ColQ1 and a catalytically inactive mutant of ColQ1 (i.e., ColQ1 E502A)^[34]^ on the larvae, we injected them with various enzyme concentrations (100 × 10^−9^−500 × 10^−9^ M). The survival of the larvae was monitored daily for eight days. Larvae injected with the catalytically inactive mutant enzyme survived (at all concentrations). In contrast, eight days after treatment with active ColQ1, the survival dropped to 0%, 20%, and 50% at concentrations of 500 × 10^−9^, 300 × 10^−9^, and 100 × 10^−9^ M enzyme, respectively (Figure S10, Supporting Information). In a next step, we examined the effects of compounds **1** and **2** on the survival of the larvae in presence of 300 × 10^−9^ M of ColQ1. Co-injection of 300 × 10^−6^ m of compound **1** increased larvae survival by 60% while 150 and 50 × 10^−6^ m concentrations showed a lower impact (~30 and 0%, Figure S11, Supporting Information). Compound **2** maintained 60% survival at 20 × 10^−6^ m until day eight while 10 × 10^−6^ and 5 × 10^−6^ m showed a lower effect (~40% and 0%) compared to the control (i.e., ColQ1) (Figure 6a).

Similar experiments were performed with *B*. *cereus* derived csn at concentrations of 35−100% (v/v). The survival of the larvae was studied for eight days after injection. After five days, only 15% of larvae injected with 100% (v/v) csn survived. With 65% and 35% (v/v) of the csn, the death of the larvae was delayed (Figure S10, Supporting Information). To investigate the effect of the collagenase inhibitors **1** and **2**, we injected the larvae with 100% (v/v) of *B*. *cereus* csn togetherwith compounds **1** (50× 10^−6^-300 × 10^−6^ m) (Figure S11, Supporting Information) or **2** (5× 10^−6^-20 × 10^−6^ m) (Figure 6b). Compound **1** at 300 × 10^−6^ m showed an increase of the survival rate from 20 to 75%, while at 150 × 10^−6^ m, the survival improved to 45% (Figure S11, Supporting Information). Compound **2** enhanced the survival from 25% to 73% at 20 × 10^−6^ m (Figure 6b). This difference between survival of larvae injected with ColQ1 and csn might be due to the high quantities of ColQ1 used in the experiment (i.e., 300 × 10^−9^ M), which is 300-fold the collagenase concertation in csn. This indicates that the action of csn on the larvae might be connected to other virulence factors (such as sphingomyelinase and non-hemolytic enterotoxins) as well as collagenase, which could work together to kill the larvae.^[63,64]^ This also suggests that both compounds might target other virulence factors in the csn, therefore further experiments could be performed in the future to confirm this.

The toxic effect exerted by *B*. *cereus* collagenases might be related to the activation of melanization mechanisms in the larvae since the dead larvae turned black, as suggested for other metalloproteases.^[65–70]^ In addition, it has been shown that collagenases digest hemolymph proteins of the larvae into small peptides, which trigger an immune response finally leading to their _death._^[65–69]^

## 3 Conclusions

Virulence factors and their inhibitors are currently gaining wide attention because of their potential to limit the evolution of antibiotic resistance and to treat infections by reducing bacterial pathogenicity.^[13]^ Therefore, full characterization of virulence factors is essential to understand their role during infection and to predict whether their inhibition is beneficial for the treatment. In the present work, we characterized the collagenolytic activity of a recently discovered recombinant *B*. *cereus* ColQ1 virulence factor^[34]^ and *B*. *cereus* csn. In addition, we evaluated the biological effects of two small molecules that inhibit collagenases of *B*. *cereus* and other pathogens. In this context, an ex vivo pig-skin model of *B*. *cereus* infection was used to investigate *B*. *cereus* collagenases and the consequences of their inhibition. This model highlights the ability of *B*. *cereus* collagenase to decompose fibrillar COLs and disrupt their regular alignment. This mechanism might lead to an accelerated bacterial infiltration and penetration into deeper sites of the host. Moreover, as previously reported, this mechanism is one of the main obstacles to the wound-healing process.^[59,73]^ We demonstrated that *B*. *cereus* csn collagenases induced cytotoxicity in fibroblasts and keratinocytes, which could be minimized using bacterial collagenase inhibitors. In an in vivo model using *G. mellonella* larvae, we showed that ColQ1 and *B*. *cereus* csn are toxic and induce the death of the larvae. Treatment with collagenase inhibitors significantly increased their survival rate. These findings provide new insights into the functions of *B*. *cereus* collagenases in wound infections and the importance of its inhibition by antivirulence, which could represent a promising therapeutic option.

## 4 Experimental Section

### Production of B. cereus ColQ1

The collagenase unit of ColQ1 from *B*. *cereus* strain Q1 (Uniprot: B9J3S4; Tyr94-Gly765) was expressed and purified as previously described.^[34]^

### B. cereus csn Production

*B*. *cereus* ATTC 14 579 strain was prepared as described before.^[35]^ *B*. *cereus* was grown in RPMI medium (+10% FCS, 1% Glutamine) (Gibco) at 30 °C ON with 160 rpm shaking. The next day, csn was harvested by centrifugation at 3000 x g for 10 min at 4 °C. The csn was sterile-filtered with 0.22 μm filter (Greiner) then, it was aliquoted and stored at −80 °C until use.

### In vitro FRET-Based Peptidolytic Assay

IC_50_ measurements were performed as previously reported.^[33]^ In short, ColQ1 was incubated with compound **2** at RT for 1 h. The reaction was initiated by the addition of 2 × 10^−6^ m of the collagenase-specific peptide substrate Mca-Ala-Gly-Pro-Pro-Gly-Pro-Dpa-Gly-Arg-NH_2_ (FS1-1; Mca = (7-methoxycoumarin-4-yl)acetyl; Dpa = *N*-3-(2,4-dinitrophenyl)-L-2,3-diaminopropionyl). The fluorescence was monitored for 2 min (excitation: 328 nm, emission: 392 nm) at 25 °C. The final concentrations were 1 × 10^−9^ M ColQ1, 250 mM HEPES pH 7.5, 400 mM NaCl, 10 mM CaCl_2_, 10× 10^−6^ m ZnCl_2_, 2× 10^−6^ m FS1-1, and 0 to 120 × 10^−6^ m compound **2**. Due to poor compound solubility, the DMSO concentration was adjusted to 5%. The percentage of enzyme inhibition was calculated in relation to a blank reference without compound added. All experiments were performed in triplicate. Limited by the solubility of the compound, the IC_50_ value could not be determined using non-linear regression, but was determined by linear regression using only data within the 40−60% inhibition range. Regression analysis was performed using GraphPad Prism 5 (Graph Pad Software, SanDiego, CA, USA). To determine the peptidolytic activity versus FS1-1 of the *B*. *cereus* csn, a similar assay as described above was performed. Csn samples were freshly thawed and used in the assay in three different concentrations (12%, 16% and 20% v/v). Samples were preincubated with buffer control or inhibitors for 30 min at RT, before the reactions were started upon addition of 2 × 10^−6^ m FS1-1. The final inhibitor concentrations were: 20 mM EDTA, 1x EDTA-free complete protease inhibitor cocktail (Roche, Woerden, The Netherlands) as serine and cysteine protease inhibitors, 1.83mMcompound **1** and 95 × 10^−6^ m compound **2** at a final DMSO concentration of 5%. All results were extrapolated to 100% v/v and inhibition rates were normalized to the uninhibited control. Experiments were performed in triplicate and are presented as means ± standard deviation.

### Gelatin Zymography

Aliquots of the *B*. *cereus* csn were loaded onto 10% SDS-PAGE gels containing 0.2% gelatin (Roth, Karlsruhe, Germany) and separated by electrophoresis at 4 °C. After separation, the gels were sliced into 4 pieces (marker lane plus 2 sample lanes) each and incubated in the respective renaturation buffer (50 mM HEPES pH 7.5, 200 mM NaCl, 10 × 10^−3^ m CaCl_2_, 10× 10^−6^ m ZnCl_2_, 2.5% Triton X-100) supplemented with (i) nothing (control), (ii) 1x EDTA-free cOmplete protease inhibitor cocktail (Roche, Woerden, The Netherlands), (iii) 300 × 10^−6^ m compound **1** or (iv) 100 × 10^−6^ m compound **2** at RT for 2×30 min with gentle agitation. The gel slices were then equilibrated in the respective developing buffer (50 mM HEPES pH 7.5, 200 mM NaCl, 10 mM CaCl2, 10 × 10^−6^ mZnCl_2_, 0.02% Brij-35) supplemented with the aforementioned compounds (i-iv) at RT for 2×10 min with gentle agitation, and then incubated on at 37 °C in fresh, supplemented developing buffer. Transparent bands of gelatinolytic activity were visualized by staining with 0.1% Coomassie brilliant blue G-250 dye ON. Gels were scanned using Chemi-Doc XRS+ imaging system (Biorad, USA) and image analysis was performed with Image Studio Lite v5.2 software (Li-Cor Biosciences, USA). The integration area of the indicated molecular weight regions was measured, and values were expressed as a ratio of the control area from the same gel (no additional treatment; set to unity). Results were thereby standardized for each gel and expressed in dimensionless units. Results were obtained from two separate experiments for each condition.

### COL Cleavage Assay

Acid-soluble type I COL from bovine tail (Thermo Fischer Scientific) at a final concentration of 1 mgmL^−1^ was digested at 25 °C by 50 ng ColQ1 in 250 mM HEPES, 150 mM NaCl, 5 mM CaCl2, 5 × 10^−6^ m ZnCl2, pH 7.5. Compounds **1** and **2** were included at different concentrations, and incubated together with COL and ColQ1 for 3 h. The reaction was stopped by the addition of 50 mM EDTA followed by visualization with 12% SDS-PAGE gels. Results were obtained from two independent experiments for each compound.

### Synthesis of Compounds 1 and 2

The synthesis was performed according to the synthetic scheme that we published before.^[32,30]^

### Docking of Compounds 1 and 2

The crystal structure of the peptidase domain of ColH (5o7e) with 1.87 Å resolution was used as target model for the docking. Ligand files were prepared as input for the docking software using OpenBabel (protonation state)71 In case of compound **2**, the thiolate derivative was used as input, as the mercaptoacetamide compound is known to hydrolyze in aqueous solution.^[33]^ The final docking was performed using the Molecular Forecaster suite.^[49]^ In short, the protein structure was prepared using the PREPARE and PROCESS modules with a ligand cutoff of 7 Å (particle water option). The ligands were prepared using the SMART module. Docking calculations were performed using FITTED. The docking software was validated via redocking the ligand 9NB, resulting in an RMSD of 0.43Å.The PyMOLMolecular Graphics System, version 2.0.6.0a0,Schrödinger,LLC,was used for generating figures.^[72]^

### ColQ1 Activity on Ex vivo Pig-Skin Model

The ex vivo pig-skin model was performed as reported earlier.^[38]^ The skin explants of 15 mm diameter were made from ears of young pigs which were provided by a local slaughterhouse. Once the ears were received, several steps of sterilization were performed. The ears were punched, washed with sterile water followed with 3 x DMEM medium containing 10% FBS, 1% Pen-Strep and 250 ng mL^−1^ amphotericin B, with a minimum of 15 min incubation time. To assess the sterilization by antibiotics, randomly selected skin punches were incubated in DMEMmedium at 37°C ON. The next day, the exposed DMEM was plated on LB-agar plate without antibiotic to check for bacterial growth. After washing the explants, they were stored at −80 °Cfor a maximum of one month in DMEM supplemented with 15% (v/v) glycerol. The storage conditions were selected based on the viability of the skin which we evaluated over one month with the MTT assay at 37 °C, −20 °C and − 80°C (Figure S3, Supporting Information). To investigate the activity of collagenase effect ex vivo, the skin samples were thawed and incubated at 37 °C for one hour in DMEM medium containing 10 × 10^−6^ mZnCl_2_ and 4 mM CaCl_2_. While the epidermal side of the skin was exposed to air, the dermal side was incubated in DMEM medium with ColQ1 or csn for several time periods. The skin was incubated with different concentrations of ColQ1 ranging from 100× 10^−9^-500 × 10^−9^ Mand of *B*. *cereus* csn (0−100%v/v)for several days in a total volume of 300μL containing ColQ1 or csn together with DMEM and tissue explant. To estimate the release of Hyp into the DMEM medium, the medium was collected and stored at −20 °C. Hyp quantification was performed using a Hydroxyproline assay kit (Sigma Aldrich).In short, Hyp was converted into a colorimetric product after adding 100μL chloramine T/oxidation buffer mixture, 100 μL4-(dimethylamino)benzaldehyde diluted in perchloric acid/isopropanol to 10 μL of DMEM medium and measured at a wavelength of 560 nm. For further evaluation, the skin tissues that were treated for 24 h were fixed with 4% paraformaldehyde (PFA) and stored at 4 °C.The fixed skin was stored ON with 10% and then 25% sucrose in PBS ON in order to prevent tissue damage before downstream evaluation. The data were plotted with GraphPad Prism 8 for three independent experiments and to calculate the probability value one-way ANOVA was performed and statistical significance was analyzed by Tukey test.

### Ex vivo Pig-Skin Model for Evaluating the Effect of ColQ1 Inhibitors

In order to select adequate inhibitor concentrations, the skin was treated with 300 × 10^−9^ M ColQ1 (optimal concentration of ColQ1 selected from the previous assay) and gradient concentrations of collagenase inhibitors **1** and **2**. A total of 12 skin punches per compound were treated in duplicate for six conditions followed by incubation at 37 °C for 24 h, 5% CO_2_ and 300 rpm. Non-treated condition was considered as a healthy state, the other samples were incubated with 300× 10^−9^ M ColQ1 combined with either compound **1** (0−400 × 10^−6^ M) or compound **2** (0−50 × 10^−6^ M). After 24 h, all samples were fixed in 4% PFA and stored after treating them with 10% and 25% sucrose/PBS as described before and prepared for microscopic and biochemical analysis. To analyze the Hyp content in the DMEM medium for each condition, the DMEM was collected before and after treatment and stored at −20 °C. Finally, the optimal inhibitor concentration was determined by microscopy and biochemical evaluation. Results of three independent experiments were plotted, mean ± standard deviation.To estimate the probability value one-way ANOVA was performed and statistical significance was analyzed to illustrate the significant differences between non-treated versus treated samples. (*** *p* ≤ 0.001).

### Sample Preparation for SHG and Immunostaining of COL Subtypes

For immunostaining, tissue samples were stained with primary antibodies. (COL I (Rabbit polyclonal anti-type I collagen, (600-401-103-0.1 Rockland); COL III (rabbit anti human collagen III antibody, (Abcam, ab7778)); COL V (rabbit anti human collagen V antibody, (Abcam, ab7046)) (1:200 dilutions in PBS) at RT for 1 h or at 4 °C ON. Next, the solution was removed, and all samples were gently washed with 3×100 μL PBS, followed by addition of 50 μL secondary antibody solution ((IgG (H+L) Highly Cross-Adsorbed; conjugated with AF647 (Abcam, A-21245)) in PBS (0.8% goat serum (Sigma-Aldrich, G9023-5ML)) and 1:5000 DAPI (Thermo Fisher)) at RT for 1 h or at 4°C ON. Samples were washed 3 x with PBS again. For each slide, a 0.17 μm thick 24mm x 60mm cover glass (Thermofisher) was placed on top of a layer of Parafilm and prepared with three evenly distributed drops of in total 60 μL FluoroMountG (Thermofisher, 00-4958-02, refractive index: 1.4).The slides were placed at one edge of the cover glass and slowly lowered towards it in a decreasing angle, from one side to the other. Even distribution of the mounting medium required some time and a sense of applying pressure, but when performed carefully, arising air bubbles were prevented or eliminated in this step. When all slides were sealed, everything was covered with a layer of parafilm. Since the polymerization of FluromountG requires constant pressure, some weight (e.g., a 1 L bottle PBS on top of a book) was applied on top of it for at least 4 h but optimally ON. Prior to imaging or storage at 4°C, all slides were cleaned using paper tissues and 70% ethanol in dH_2_O to remove dirt and redundant mounting medium.

### SHG and Epifluorescence Microscopy

COL fibres in the tissue were visualized using SHG generated by a Zeiss LSM 880 confocal microscope with a two-photon femtosecond pulsed laser (Chameleon Vision I, Coher-ent, Santa Clara, CA (USA)) set at 900 nm wavelength for excitation. The emitted fluorescent signal was detected before the pinhole using Zeiss Big.2 non-descanned NDD detectors in combination with a 380−430 nm band pass filter. Images were obtained using 8% laser power, with a pixel dwell time of 8.24 μs with 4 x averaging, and the detector gain set at 500. The resulting image had a size of 512×512 pixels with a pixel size of 1.38 μm. Images were taken with a Plan-Apochromat 20x/0.8 NA objective in the dermal region of the skin. Z-stack imaging was performed by selection of a representative spot in the plane with the highest SHG signal, followed by defining the first and a last plane, resulting in a Z-stack with 10 slices spanning 45 μm. Maximum intensity projections were then generated in ImageJ using the Z-project function.

Epifluorescence imaging was performed using a Nikon-Ti Eclipse inverted microscope coupled with a Lumencor SOLA white light lamp for epifluorescence. Images were captured using an Andor Clara DR-5434 camera, with filtercubes for DAPI at 365 nm staining the nuclei and the secondary antibody AF647 conjugate, which labeled COL antibodies at 640 nm. To get a good view throughout the whole skin thickness, large images with a scan area of 2×1 fields of view (10%overlapping) were captured using the Perfect Focus System. Parameters such as light intensity, exposure time, magnification, and tile scan area were adjusted individually for each COL type antibody. Thus, only treated and non-treated samples for one particular COL type immunostaining can be directly compared. For illustration purposes, a LUT threshold for each subtype was selected with the non-treated control of each condition and applied on all images of the related subtype. For a summary of the imaging conditions used, please see Table S1, Supporting Information. Triplicates of all samples were measured.

### In vitro Cell-Based Assay

NHDF (Promo Cell C-12302) and Ha-CaT (ATCC® PCS-200-011) were purchased from commercial suppliers. 50000 cells per well of NHDF and HaCaT were seeded in 96-well plate (Greiner) with DMEM medium (Gibco) including 10% (v/v) fetal bovine serum (FBS, Gibco) and 1% (v/v) Penicillin-Streptomycin (Pen-Strep) antibiotic. The cells were incubated at 37 °C for 24 h with 5% CO_2_ prior to the treatment. Next, cells were incubated with varying amounts of *B*. *cereus* csn (0-15%) in a total volume of 200 μL containing csn, cells, DMEM. To inhibit the collagenolytic activity of *B*. *cereus* csn compounds **1** and **2** were added to the culture along with 1.25% (v/v) *B*. *cereus* csn having 1% DMSO and incubated for 24 h. On the next day, cell viability was evaluated using MTT and live/dead staining assays. The MTT assay is based on the reduction of tetrazolium dye to purple insoluble formazan by mitochondrial succinate dehydrogenase. Live/dead imaging depends on staining the live cells with fluorescein diacetate (FDA) and dead cells with propidium iodide (PI). The MTT assay and live/dead staining were performed after 24 h and 48 h incubation for csn treatment and 24 h incubation after collagenase inhibitor treatment. To conduct the MTT assay, we removed the medium and washed the cells 2 x with sterile PBS buffer. Afterwards, we added 200 μL of a mixture containing fresh DMEM and 5 mgmL^−1^ MTT reagent in each well and incubated the plate for 2 h at 37 °C with 5% CO_2_. After the incubation, the medium was removed, and 200 μL of 100% DMSO was added to each well to dissolve the formazan crystals, and the plate was incubated at 37 °C for 30 min. Finally, the absorbance was measured using a PHERAstar plate reader (BMG Labtech, Ortenberg, Germany) at 0 nm for samples and at 620 nm for blanks with DMEMmedium. The viability was also evaluated via epifluorescence microscope (Leica Microsystems CMS GmbH, Wetzlar, Germany) after the live/dead staining. Cells were seeded and treated with *B*. *cereus* csn similar to the procedures mentioned above and washed 3 x with sterile PBS. 0.03 mg mL^−1^ FDA and 0.02 mgmL^−1^ PI were added into each well and incubated at 37 °C for 5 min and 5% CO_2._ Then the viability and morphology of cells were investigated with 5x magnification to obtain an overview of the quantity of live and dead cells. The morphological changes between the non-treated cells and cells treated with the csn, treated with csn was captured at bright field channel with 20x. The viability of the cells was calculated relative to non-treated controls using ImageJ Fiji software, the results were plotted with GraphPad Prism 8 for three independent experiments for each cell type and 9 images for each condition. To calculate the probability value one-way ANOVA was performed and statistical significance was analyzed by Tukey test. For display purpose, the brightness and contrast were adjusted for each image based on the values of the control image where no treatment was applied.

### Galleria mellonella Virulence Assay

*Galleria mellonella* larvae (Tru-Larv) were purchased from BioSystems Technology (Exeter, United Kingdom). Injections were performed using a LA120 syringe pump (Landgraf Laborsysteme, Langenhagen, Germany) equipped with 1 mL Injekt-F tuberculin syringes (B. Braun, Melsungen, Germany) and Sterican 0.30 × 12 mm, 30G × 1.5 needles (B. Braun). The larvae were injected in the right proleg with 10 μL of different solutions (i.e., various concentrations of *B*. *cereus* csn or ColQ1 or with only PBS). Based on that they were classified into different groups according to the following description: untreated group, treated with sterile PBS group, treated with different amount of *B*. *cereus* csn (which was diluted in sterile PBS), treated with ColQ1 diluted with sterile PBS, treated group with a mixture of 100% *B*. *cereus* csn or 300 × 10^−9^ M ColQ1 and various concentrations of compounds **1** or **2** and treated group with only one of the compounds (diluted in PBS) to evaluate the toxicity level. We considered the larvae dead if they did not move and had a black color which reflected the activation of the melanization cascade due to the toxic effect induced by virulence factors. The survival of the larvae was analyzed using GraphPad Prism 8 using Kaplan-Meier analysis followed by equality test called log-rank test. The data of three independent experiments were combined and plotted in the survival curve, 45 larvae in total were included to test compounds with the csn and 30 larvae to test compounds with ColQ1 in the three experiments.

### Statistical Analysis

Graphical data in the manuscript are communicated as the means ± SDs. Statistical comparisons were performed by Tukey one-way ANOVA test, which shows significant differences between conditions. Paramateric/non-paramateric statistical analysis used in the study were based on normality and homogeneity of variance. A value of *p* ≤ 0.001 was considered statistically significant while *p* > 0.05 was considered non-significant. The normalized measurements were statistically compared between treated and non-treated groups using generalized estimating equations model to account for correlated data arising from repeated measures. The survival of *G. mellonella* was analyzed using the Kaplan-Meier method and log-rank test was applied to calculate the significant difference between conditions.

## Supplementary Material

Supplementary material

## Data Availability

The data that support the findings of this study are available in the supplementary material of this article.
