# Supplementary material for: Inhibition of Collagenase Q1 of Bacillus cereus as a Novel Antivirulence Strategy for the Treatment of Skin-Wound Infections
Source: Adv Ther (Weinh). Author manuscript; Available in PMC 2022 Mar 18. (PMC7612511; doi:10.1002/adtp.202100222)
Supplement: Supplementary material [file EMS141010-supplement-Supplementary_material.pdf]

Supporting Information

Inhibition of collagenas Q1 of *Bacillus cereus* as a novel antivirulence strategy for the treatment of skin wound infections

Alaa Alhayek[1,2], Essak S. Khan[3,4], Esther Schönauer[5], Tobias Däinghaus[3], Roya Shafiei[1], Katrin Voos[6], Mitchell K. L. Han[3,4], Christian Ducho[6], Gernot Posselt[5], Silja Wessler[5], Hans Brandstetter[5], Jörg Haupenthal[1], Aránzazu del Campo[3,4], and Anna K. H. Hirsch[1,2,]*

(1) A. Alhayek, R. Shafiei, Dr. J. Haupenthal, Prof. Dr. A. K. H. Hirsch
Department of Drug Design and Optimization
Helmholtz Institute for Pharmaceutical Research Saarland (HIPS)
Helmholtz Centre for Infection Research (HZI)
(2) A. Alhayek, Prof. Dr. A. K. H. Hirsch
Department of Pharmacy, Saarland University, Campus, Saarbrücken, GERMANY
Email: anna.hirsch@helmholtz-hips.de
(3) T. Däinghaus, Dr. E. S. Khan, Prof. Dr. A. d. Campo
Leibniz Institute for New Materials (INM)
Chemistry Department, Saarland University, Campus, Saarbrücken, GERMANY
(4) Dr. E. Schönauer, Dr. G. Posselt, Prof. Dr. S. Wessler, Prof. Dr. H. Brandstetter
Department of Biosciences
University of Salzburg, Hellbrunner Str., Salzburg, AUSTRIA
(5) K. Voos, Prof. Dr. C. Ducho
Department of Pharmacy, Pharmaceutical and Medicinal Chemistry
Saarland University, Campus, Saarbrücken, GERMANY

Table of Contents

***Supplementary figures*...S3–S12**

***Supplementary tables*...S14**

***References*..S14**

Supplementary figures

Peptidolytic activity of the *B. cereus* csn (Figure S1)

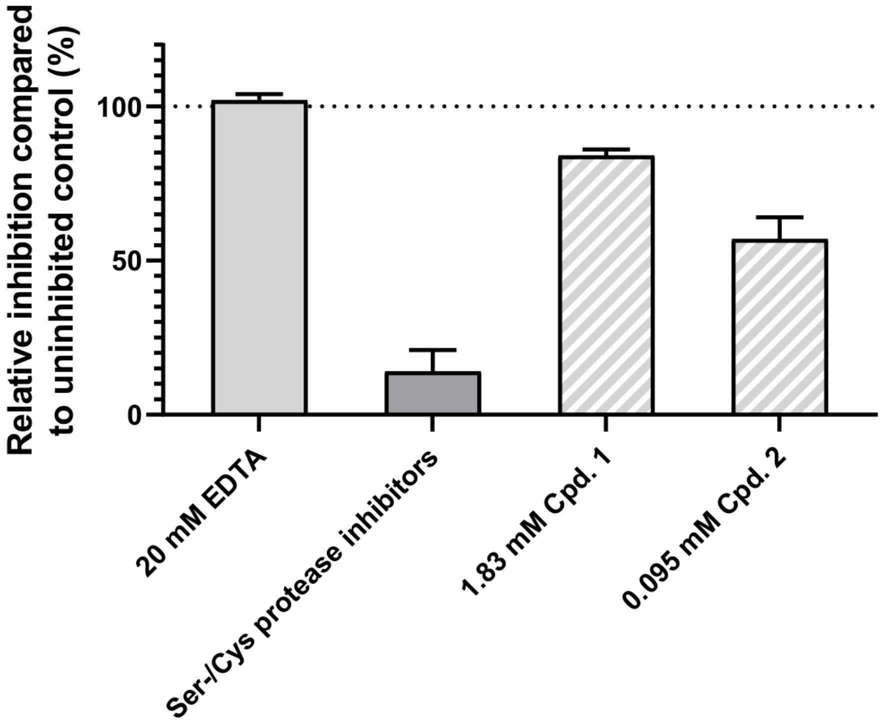

Figure S1. *B. cereus* csn activity in a collagenase-specific peptidic assay. Peptidolytic activity towards a collagenase-specific substrate by csn from *B. cereus* ATCC 14579 and inhibited with compounds **1** and **2** (n = 3, results are shown as mean ± standard deviation). Compound **1** was used at a concentration of 10x its IC_{50} vs ColQ1. Due to limited solubility in the reaction buffer, compound **2** could only be tested at a concentration of 1x IC_{50}. *B. cereus*: *Bacillus cereus*, csn: culture supernatant.

Standard curve of ColQ1 (Figure S2)

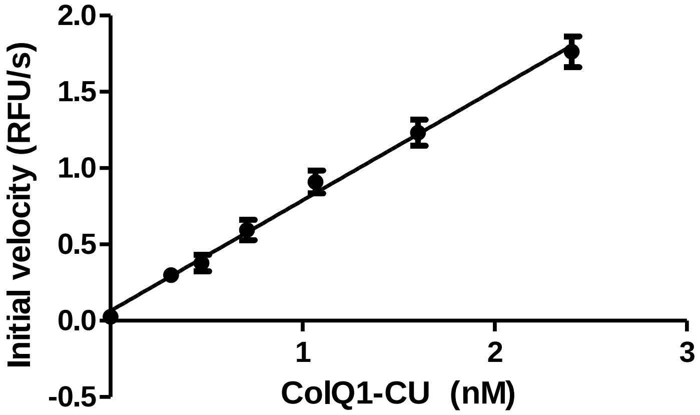

Figure S2. Representative standard curve of peptidolytic activity of ColQ1 in presence of serine and cysteine inhibitors. A 2.4 nM/ml stock solution of ColQ1 was serially diluted in the reaction buffer of the *in vitro* FRET-based peptidolytic assay. The reactions were initiated by the addition of 2 µM FS1-1 and the reactions monitored for 2 min (excitation: 328 nm, emission: 392 nm) at 25 °C. Initial velocities were calculated via linear regression. All experiments were performed in triplicates and results are shown as mean ± standard deviation.

Dose-response curves for compound 1 and compound 2 vs ColQ1 (Figure S3)

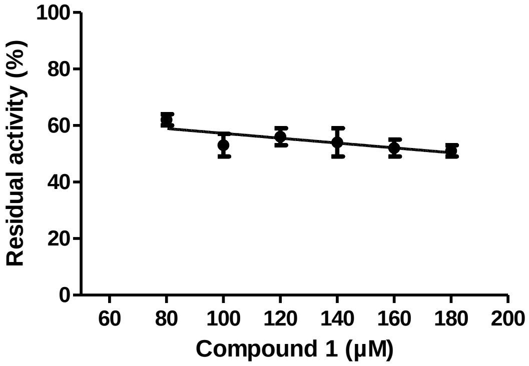

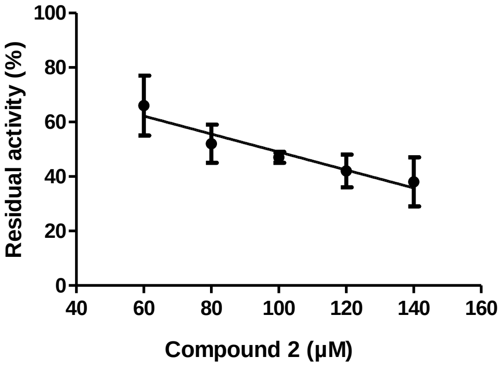

Figure S3. Representative dose-response curve for the IC_{50} value determination of compound 1 and 2 on ColQ1. Due to limited compound solubility, the IC50 determination had to be performed via linear regression and was limited to the linear portion of the sigmoidal response curve.[1] The compounds were preincubated for 1 h at RT before initiating the reaction by addition of 2 µM FS1-1. The reactions were monitored for 2 min (excitation: 328 nm, emission: 392 nm) at 25 °C. Initial velocities were calculated via linear regression and normalized to a non-inhibited control reaction. All experiments were performed in triplicates and results are shown as mean ± standard deviation.

Bright-field and DAPI signals of the tissue used for the immunostaining of fibrillar collagens (Figure S4)

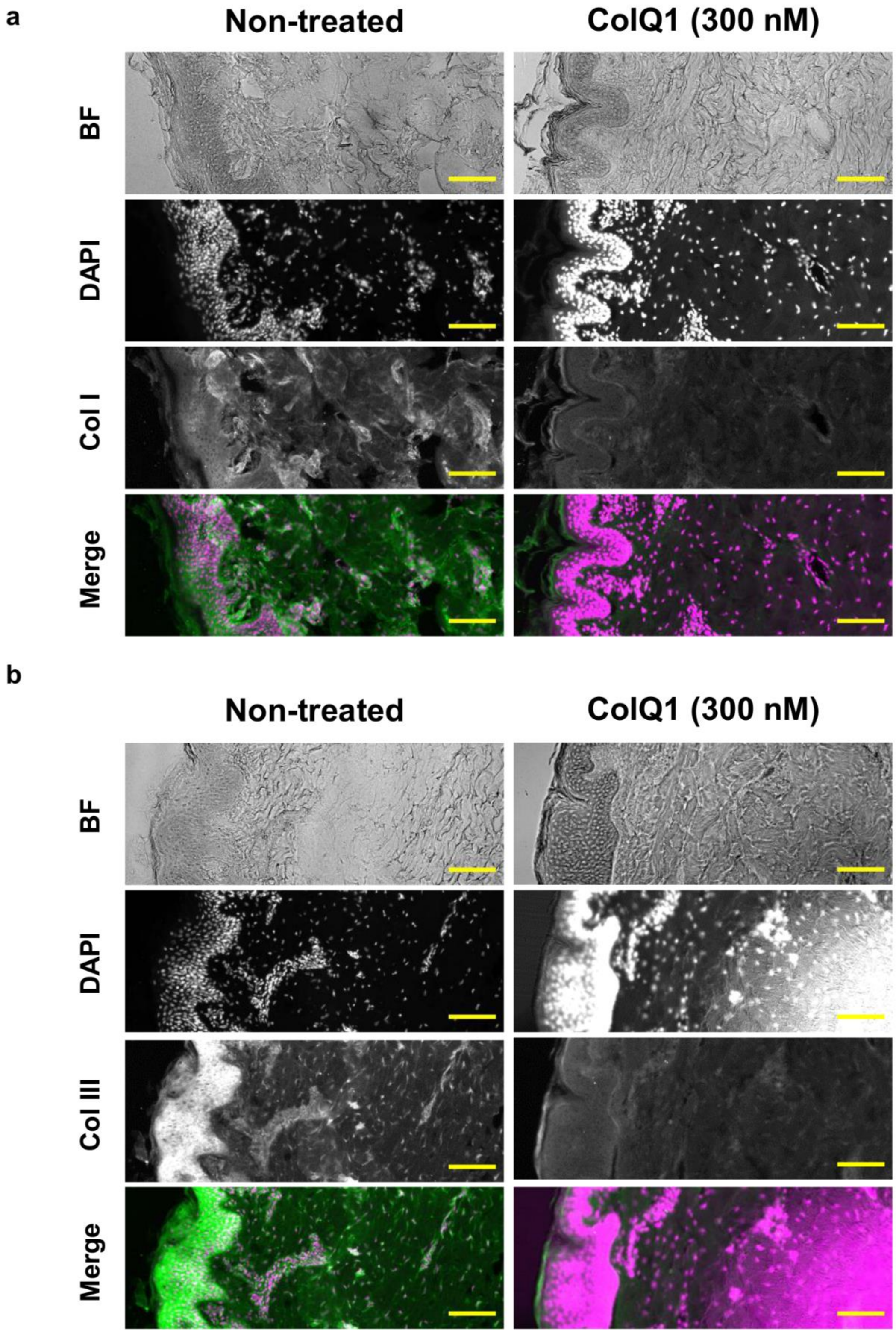

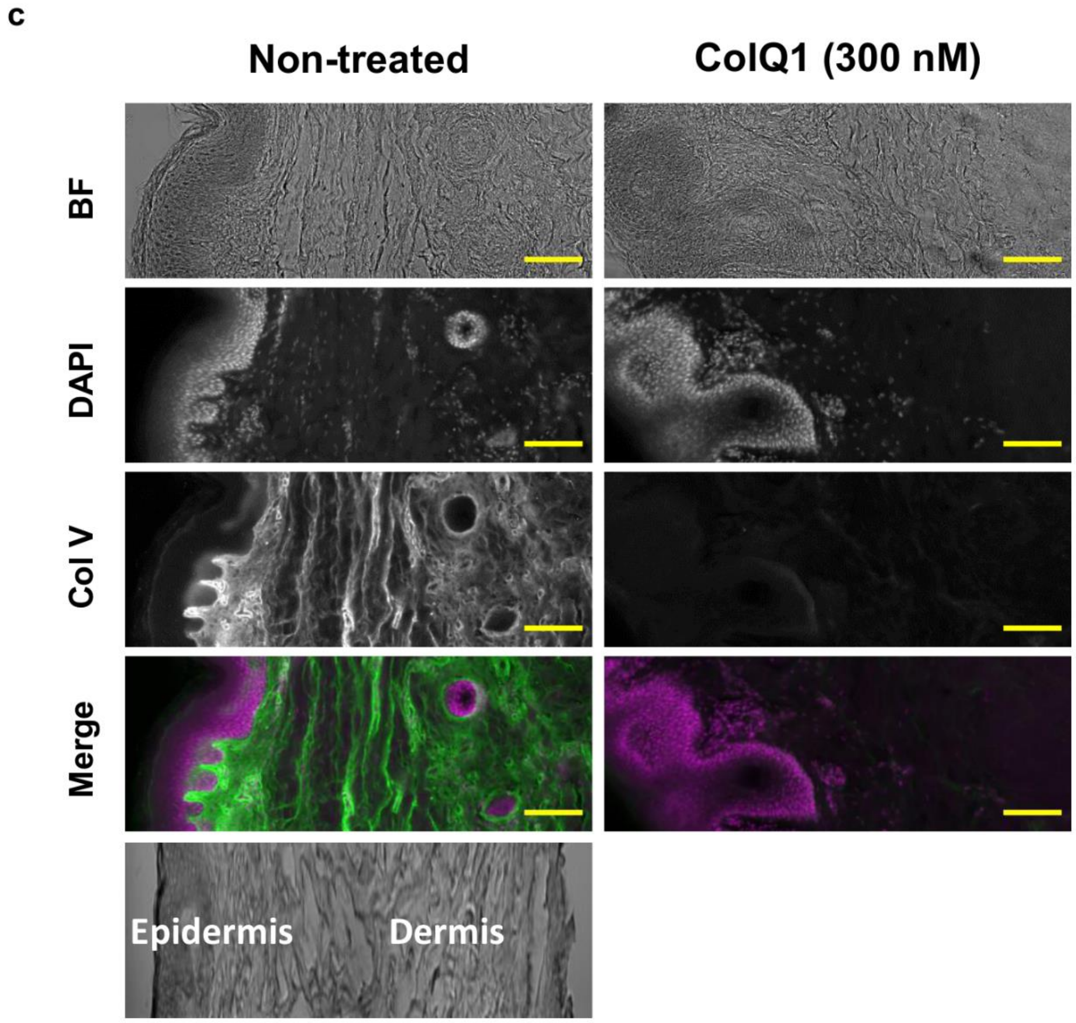

Figure S4. Bright-field and DAPI images of the non-treated tissue and ColQ1-treated tissue (a) COL I (Collagen I), **(b)** COL III (collagen III), **(c)** COL V (collagen V). Dermal and epidermal regions are labeled. Scale bar: 100 µm.

Effect of storage conditions on skin tissue (Figure S5)

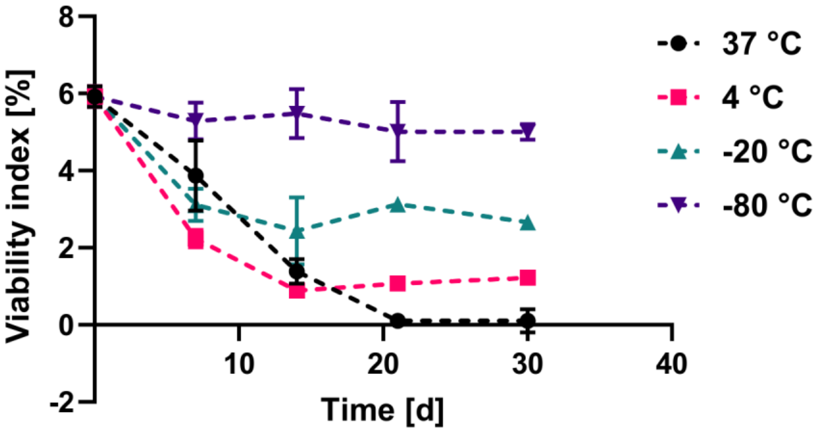

Figure S5. The influence of storage temperature on skin viability.

SHG and fibrillar collagen imaging for the tissue challenged with ColQ1 with or without compound 1 (Figure S6)

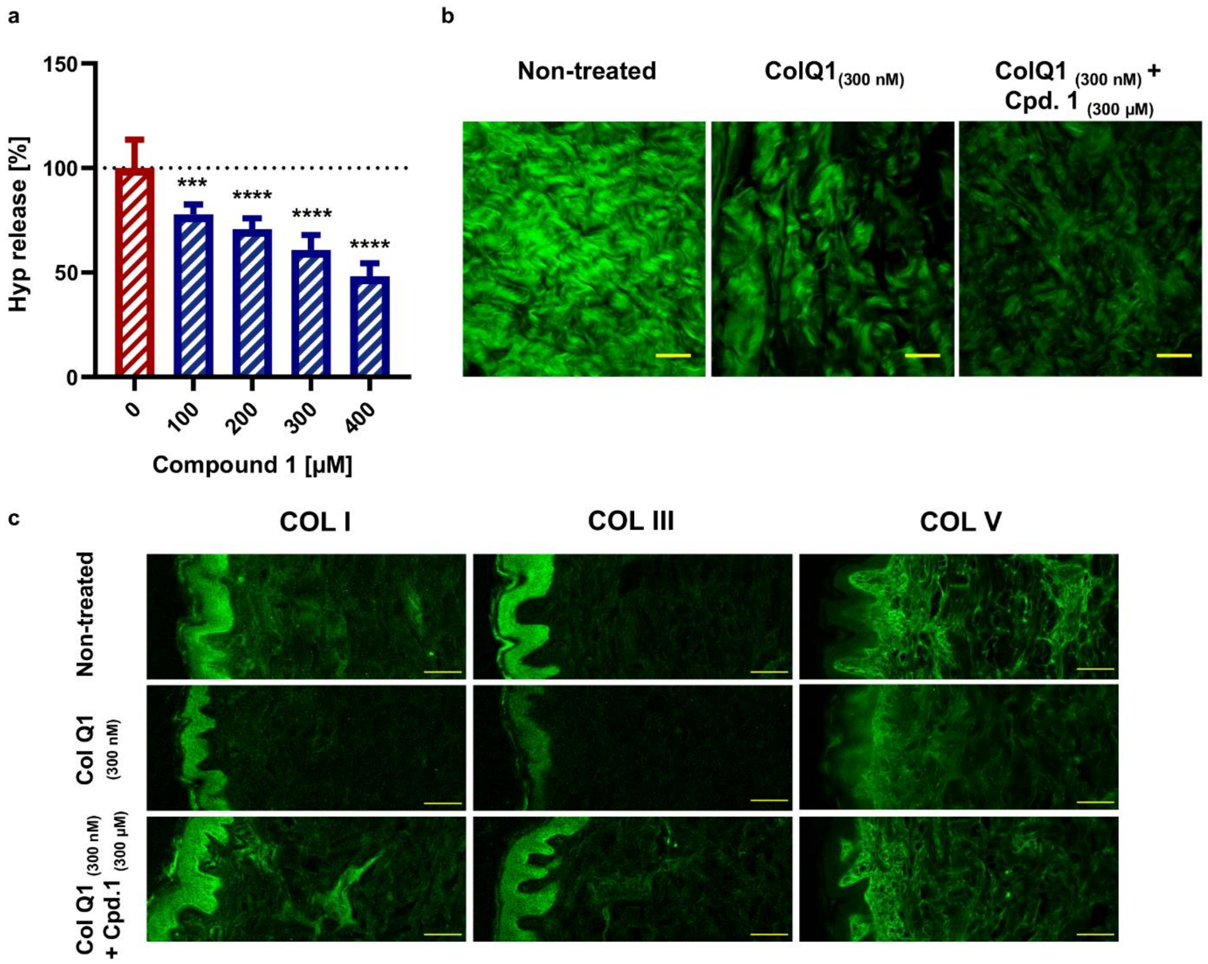

Figure S6. Compound 1 inhibited the collagenolytic effect of ColQ1 *ex vivo* in skin tissue. (a) Dose-dependent effect of compound **1** in Hyp release assay. **(b)** Confocal SHG images revealed an improved collagen signal with 300 μM of compound **1** (tissue challenged with 300 nM ColQ1) compared with 300 nM ColQ1 without inhibitor. **(c)** Immunostaining of fibrillar COLs of the non-treated skin and treated with ColQ1 with or without compound **1**. Statistical analysis was performed with one-way ANOVA and statistical significance was analyzed by Tukey test. Significance was calculated by comparing non-treated *vs* treated tissue with compound **1** (mean ± SD, **** $p \leq 0.0001$, *** $p \leq 0.001$). Hyp: hydroxyproline, COLs: collagens, SHG: second-harmonic generation. Scale bar: 100 μm for SHG images and for the immunostained images.

Viability and imaging data of fibroblast and keratinocyte cells challenged with *B. cereus* csn (Figure S7–S9)

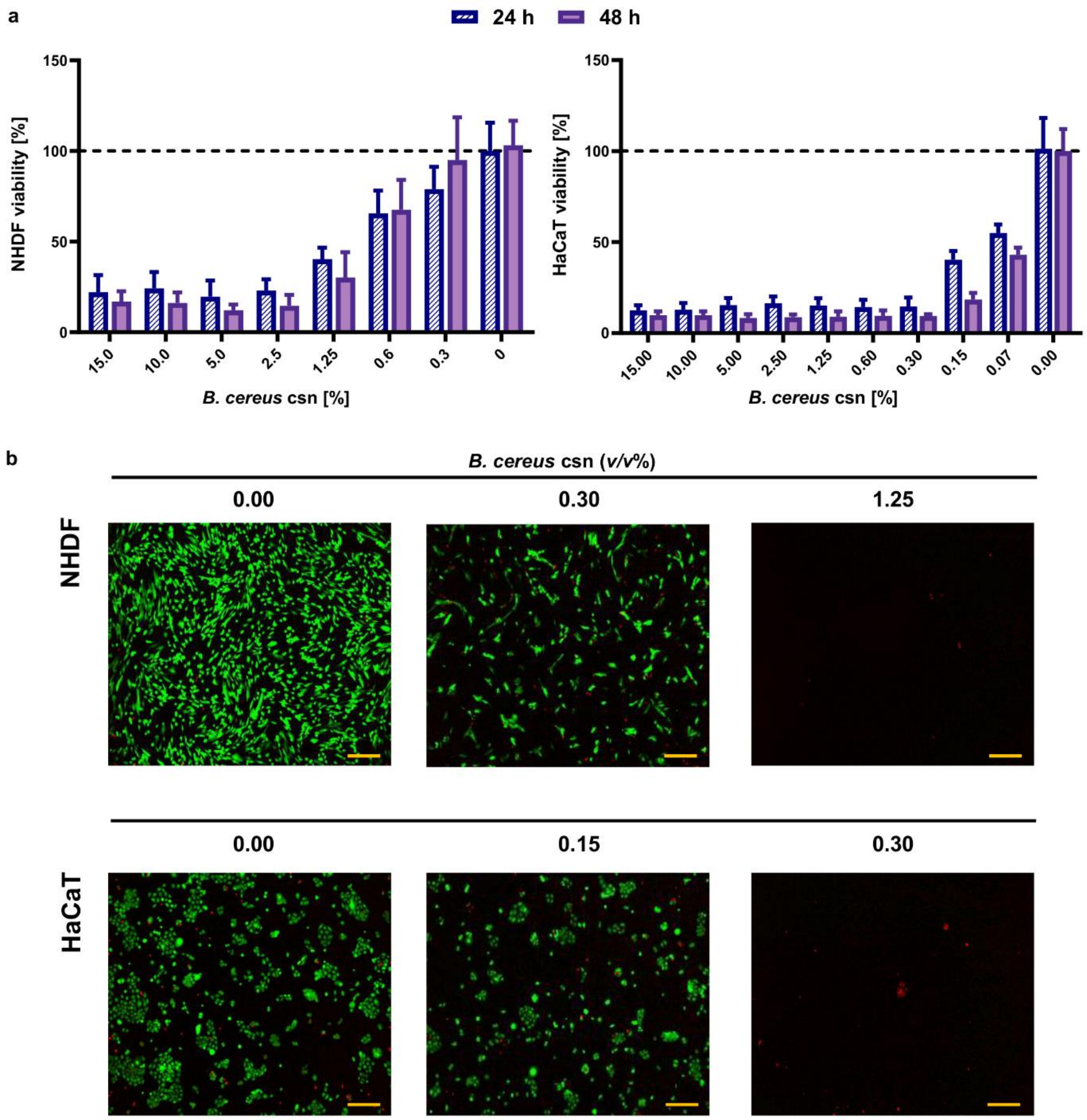

Figure S7. Viability data of fibroblast (NHDF) and keratinocyte (HaCaT) cells upon the treatment with *B. cereus* csn. (a) Representations of MTT data of skin cells after 1 and 2 days of incubation with various concentrations of *B. cereus* csn. **(b)** Composite signal of live and dead skin cells challenged with various concentrations of *B. cereus*. Green signals: living cells and red signals: dead cells, red signal in some cases was lost because the detached cells were washed away after the rinsing step with PBS. (n = 3, results are shown as mean ± standard deviation), *B. cereus*: *Bacillus cereus*, csn: culture supernatant. Scale bar: 200 µm.

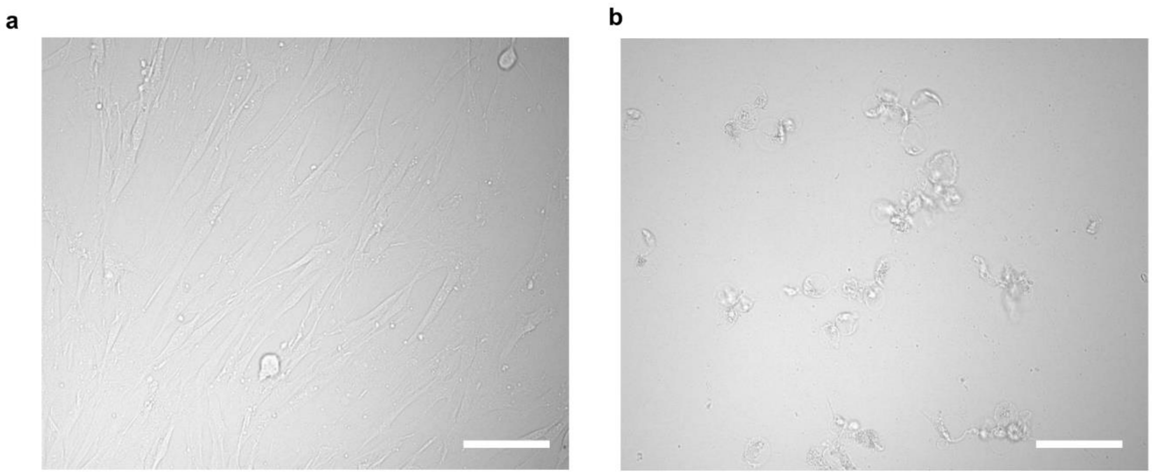

Figure S8. Bright-field signals of skin cells challenged with *B. cereus* csn visualized with 20X objective. (a) Non-treated NHDF cells compared with **(b)** NHDF cells treated with 1.25% (*v/v*) *B. cereus* csn. *B. cereus: Bacillus cereus*, csn: culture supernatant Scale bar: 100 µm

Live/dead imaging of the effect of compounds 1 and 2 on skin cells challenged with *B. cereus* csn (Figure S9)

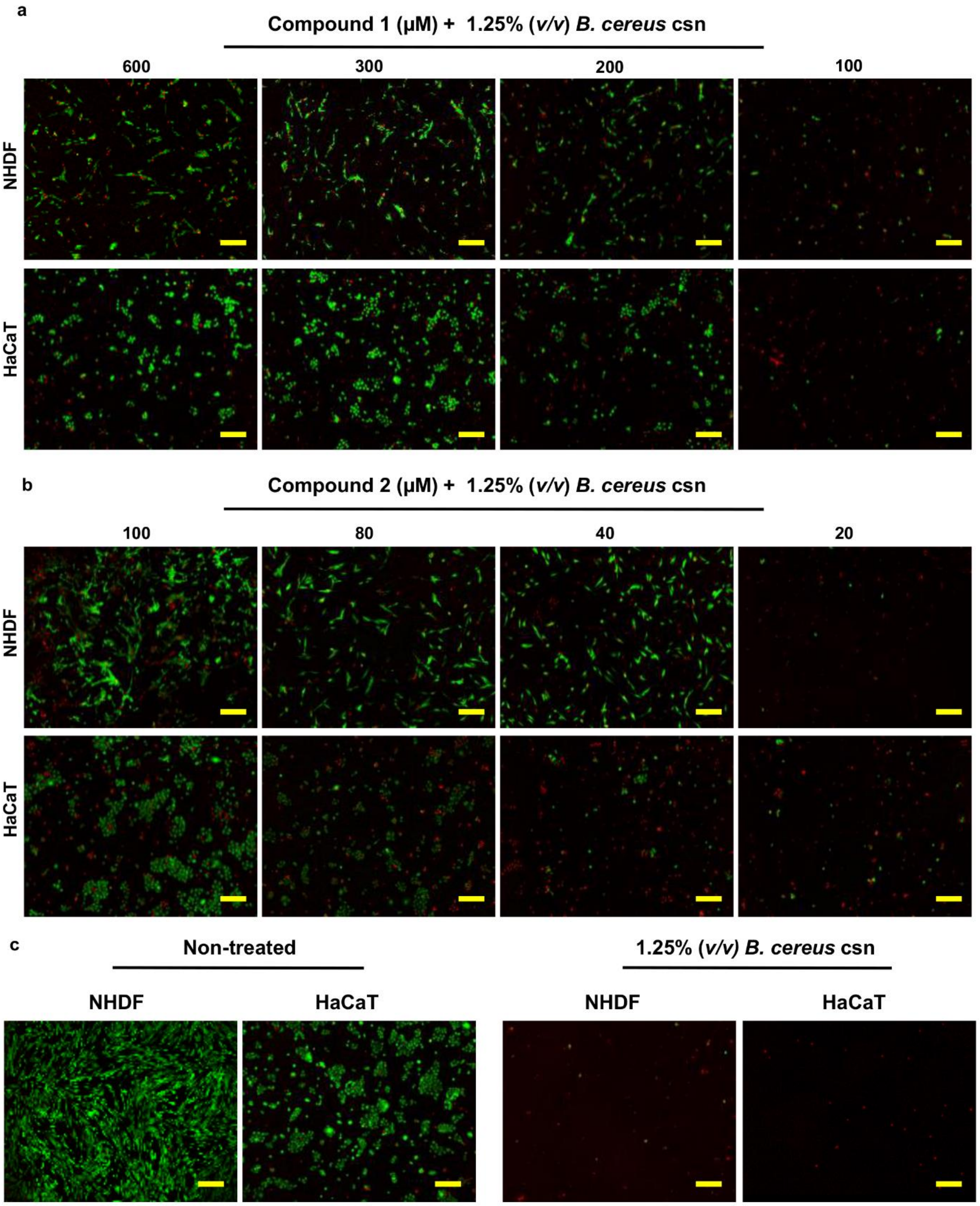

Figure S9. Compounds 1 and 2 maintained the viability of skin cells upon the treatment with 1.25% (*v/v*) of *B. cereus* csn. Live/dead imaging with fibroblasts (NHDF) and keratinocytes (HaCaT) challenged with 1.25% (*v/v*) of *B. cereus* csn with and without compounds **(a) 1** and **(b) 2** and **(c)** non-treated cells. Green signals: living cells and red signals: dead cells, red signals in some cases were lost because the detached cells were washed away after the rinsing step with PBS. *B. cereus: Bacillus cereus*, csn: culture supernatant. Scale bar: 200 µm for images.

G. mellonella survival analysis of the larvae challenged with _B. cereus_ csn or with ColQ1 (with or without compound 1) and its inactive mutant version (Figure S10–S11)

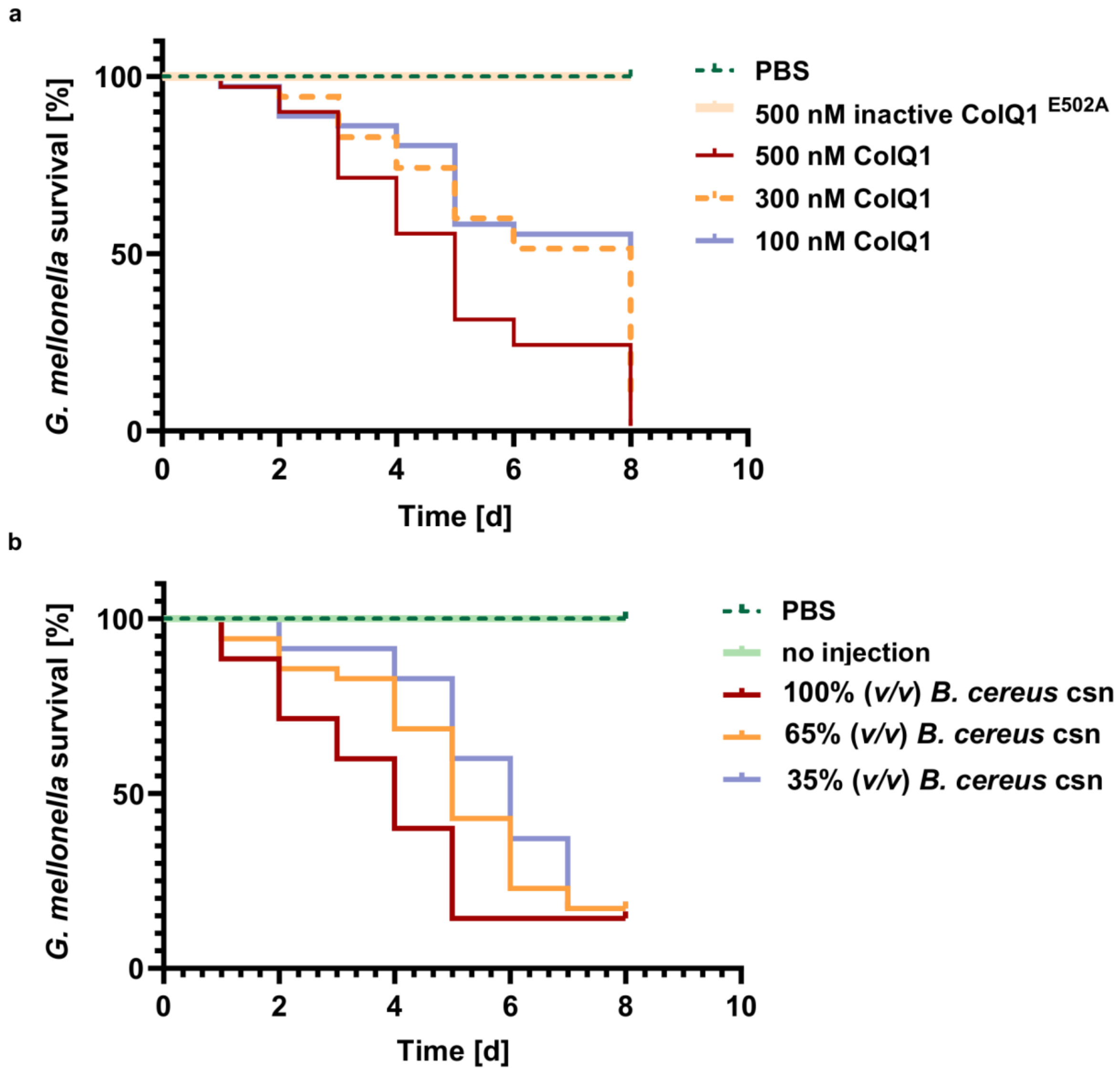

Figure S11. Probability of survival of the _Galleria mellonella_ larvae treated _B. cereus_ csn or ColQ1. (a) The survival of larvae challenged with different concentrations of ColQ1 (active and inactive mutant), the survival rate of larvae treated with 300 and 100 µM of the inactive mutant ColQ1 E502A was 100%. **(b)** The survival of larvae challenged with different concentrations of *B. cereus* csn. *B. cereus*: *Bacillus cereus*, csn: culture supernatant. Each curve represents results of three independent experiments.

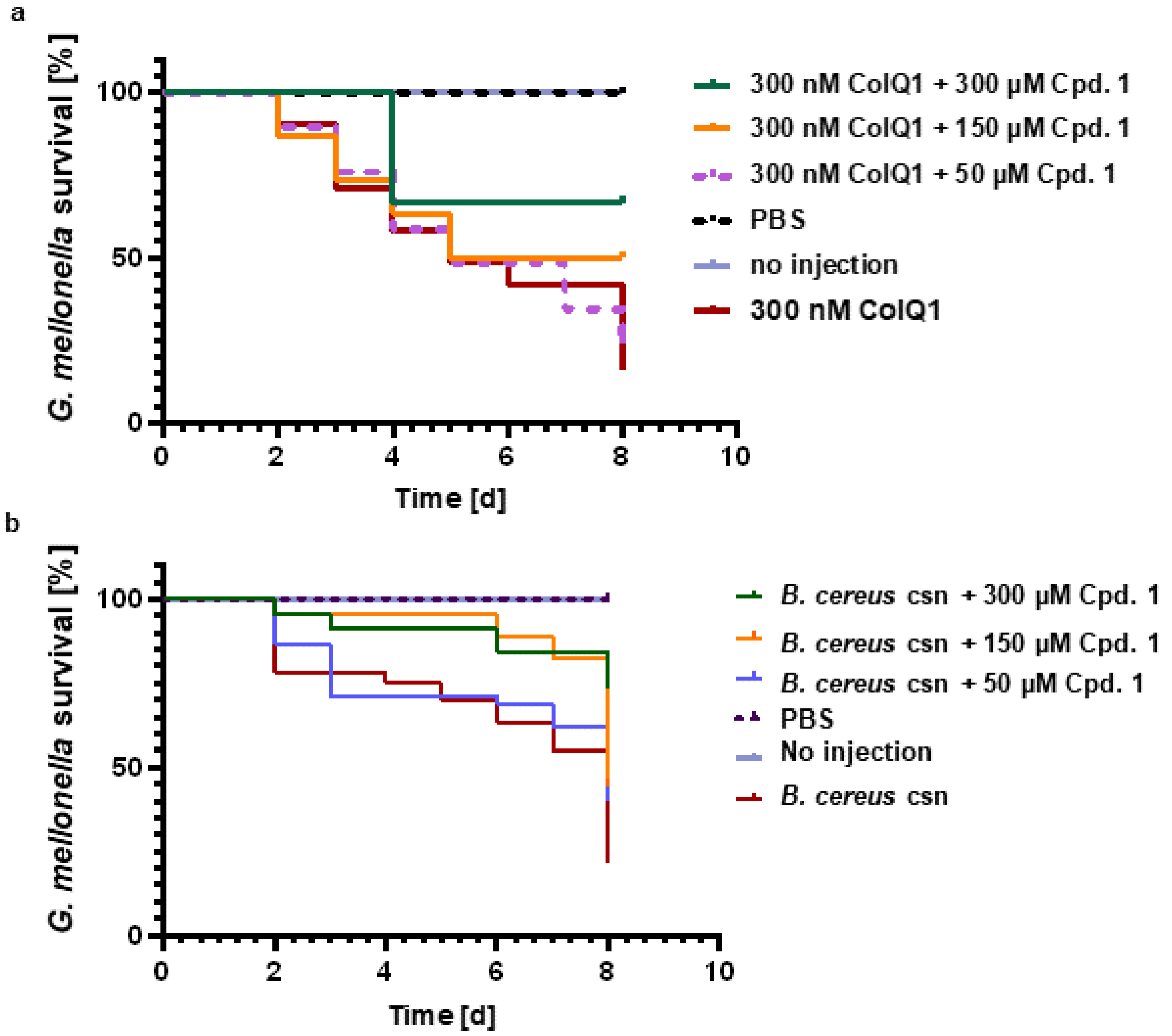

Figure S11. Kaplan–Meier survival analysis of larvae treated with B. cereus csn with and without compound 1. (a) Survival analysis of larvae treated with 300 nM ColQ1 and with various concentrations (50–300 µM) compound **1**. **(b)** The improvement in the survival of larvae challenged with 100% (*v/v*) *B. cereus* csn and various concentrations of compound **1** (50–300 µM). The statistical difference between groups treated with 300, 150, and 50 µM of compound **1** and treated with only 300 nM ColQ1 is $p = 0.0002$, $p = 0.0510$, and $p = 0.7593$, sequentially (log-rank). The statistical difference between groups treated with 300, 150, and 50 µM of compound **1** and treated with only 100% (*v/v*) *B. cereus* csn is $p < 0.0001$, $p = 0.0096$, and $p = 0.0107$, respectively. The survival rate for the larvae treated with compound **1** in PBS was 100%. *B. cereus: Bacillus cereus*, csn: culture supernatant.

Supplementary tables

Table S1. Summary of epifluorescence imaging conditions

**Imaging conditions Collagen**	**Objective**	**Exposure time**	**LUT settings (Min–Max)**
**Fig 1 – ColQ1 effect**			
**COL I**	Plan Apo λ 10x	700 ms	500–1200
**COL III**	Plan Apo λ 10x	700 ms	500–1200
**COL V**	SPlanFluor 20x LWD Dry	200 ms	500–5600
**Fig 3 – (compound 2)**			
**COL I**	SPlanFluor 20x LWD Dry	200 ms	500–8000
**COL III**	SPlanFluor 20x LWD Dry	200 ms	500–1400
**COL V**	SPlanFluor 20x LWD Dry	1 s	500–1600
**S4 Fig – (compound 1)**			
**COL I**	SPlanFluor 20x ELWD DIC N1	200 ms	500–560
**COL III**	SPlanFluor 20x ELWD DIC N1	200 ms	500–560
**COL V**	SPlanFluor 20x ELWD DIC N1	1 s	500–800

References

1. Sebaugh, J. L. & McCray, P. D. Defining the linear portion of a sigmoid-shaped curve: bend points. *Pharm. Stat.* **2**, 167–174 (2003).
